# Targeting Ovarian Cancer with Chalcone Derivatives: Cytotoxicity and Apoptosis Induction in HGSOC Cells

**DOI:** 10.3390/molecules28237777

**Published:** 2023-11-25

**Authors:** Elif Merve Aydin, İdil Su Canıtez, Eleonora Colombo, Salvatore Princiotto, Daniele Passarella, Sabrina Dallavalle, Michael S. Christodoulou, Irem Durmaz Şahin

**Affiliations:** 1 Koç University Research Center for Translational Medicine (KUTTAM), Istanbul 34450, Turkey; 2 Dipartimento di Chimica, Università degli Studi di Milano, 20133 Milano, Italy; 3 Ann Romney Center for Neurologic Diseases, Department of Neurology, Brigham and Women’s Hospital and Harvard Medical School, Boston, MA 02115, USA; 4 Department of Food, Environmental and Nutritional Sciences (DeFENS), University of Milan, Via Celoria 2, 20133 Milan, Italy; 5 School of Medicine, Koç University, Istanbul 34450, Turkey

**Keywords:** chalcones, ovarian cancer, OVCAR-3, OVSAHO, KURAMOCHI

## Abstract

Ovarian cancer ranks as the eighth most prevalent form of cancer in women across the globe and stands as the third most frequent gynecological cancer, following cervical and endometrial cancers. Given its resistance to standard chemotherapy and high recurrence rates, there is an urgent imperative to discover novel compounds with potential as chemotherapeutic agents for treating ovarian cancer. Chalcones exhibit a wide array of biological properties, with a particular focus on their anti-cancer activities. In this research, we documented the synthesis and in vitro study of a small library of chalcone derivatives designed for use against high-grade serous ovarian cancer (HGSOC) cell lines, specifically OVCAR-3, OVSAHO, and KURAMOCHI. Our findings revealed that three of these compounds exhibited cytotoxic and anti-proliferative effects against all the tested HGSOC cell lines, achieving IC_50_ concentrations lower than 25 µM. Further investigations disclosed that these chalcones prompted an increase in the subG1 phase cell cycle and induced apoptosis in OVCAR-3 cells. In summary, our study underscores the potential of chalcones as promising agents for the treatment of ovarian cancer.

## 1. Introduction

Ovarian cancer is one of the most lethal gynecologic cancers in women worldwide [1]. In 2020, 314,000 new ovarian cancer patients were diagnosed, and 207,000 death cases occurred worldwide. It is estimated that there will be 446,000 new cases and 314,000 deaths in 2040 [2]. The histological subtypes of epithelial ovarian cancer are low- and high-grade serous, clear cell, mucinous, and endometrioid tumors. Among these subtypes, high-grade serous ovarian cancer (HGSOC) is the most malignant type, and the recurrence rate of the patients is high [3]. Most HGSOC patients are diagnosed at advanced stages due to unspecific symptoms of the disease and a lack of tumor markers for diagnosis [4].

Although the treatment options vary according to the stage of cancer, cytoreductive surgery and follow-up adjuvant chemotherapy is the most common treatment method for HGSOC [5]. Platinum-based drugs (carboplatin and cisplatin) are used for first-line adjuvant chemotherapy for HGSOC. However, most patients acquire drug resistance after first-line therapy [6,7]. Thus, based on patient recurrence status, poly-(ADP-ribose) polymerase inhibitor (PARPi) drugs (olaparib and Niraparib) are used as second-line therapy for HGSOC patients [8]. PARPi function is based on the concept of synthetic lethality, including double-strand break (DSB) and displaying targeted cell death towards ovarian cancer cells that are deficient in homologous recombination repair mechanism-related genes [9]. Despite its notable achievements, the effect of PARPi is limited due to innate and acquired drug resistance phenomena [10]. Poor prognosis is associated with a high recurrence rate at 3 years (>75%) in HGSOC patients after first-line and second-line chemotherapy. Thus, it is important to investigate new treatment strategies to enhance the survival rate of high-grade serous ovarian cancer patients [11].

Chalcones are precursors of flavonoids, which are common chemical structures found in nature, and plant extracts are the most important sources of chalcones [12]. They show several important biological properties, including anti-microbial, anti-fungal, anti-inflammatory, anti-diabetic, and, most importantly, anti-cancer [13,14]. They act on targets that normally play roles in cancer progression and are involved in important cellular pathways such as cell cycle, angiogenesis, and multidrug resistance [15]. In the literature, chalcones were shown to have in vitro and in vivo anti-cancer activities against different cancer types [16,17,18,19,20]. Recently, we showed that chalcone derivatives can be MAO-B-selective inhibitors for the treatment of neurodegenerative diseases [21] and demonstrated high cytotoxic activity against liver cancer cells [16] inducing SubG1/G1 arrest and apoptosis, demonstrating that chalcone derivatives can be considered as potential new agents for liver cancer treatment.

In this context, we envisioned that the promising scaffold of chalcones could be used in the treatment of ovarian cancer, and it could be an alternative treatment option to overcome drug resistance in HGSOC [22]. In this study, we prepared a small library of chalcone derivatives containing electron-withdrawing or electron-donating substituents on both aromatic rings and examined their anti-cancer properties in high-grade serous ovarian cancer cells.

## 2. Results and Discussion

### 2.1. Chemistry

**Scheme 1:**
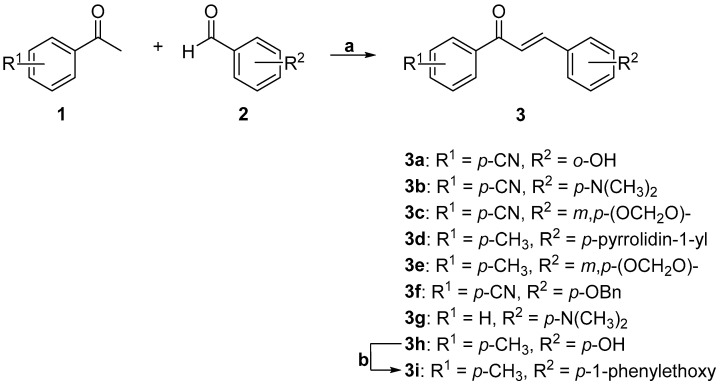
Reagents and conditions: (a) NaOH (30%), MeOH, r.t.; (b) (1-bromoethyl)benzene, K_2_CO_3_, acetone, reflux.

The small collection of chalcone derivatives was synthesized using the Claisen−Schmidt condensation methodology previously described [16]. Briefly, an aqueous solution of sodium hydroxide was added slowly to a methanol solution of the appropriate acetophenone (**1**). After the solution was cooled to room temperature, the appropriate benzaldehyde (**2**) was added. The mixture was stirred at room temperature for 12 h to provide the corresponding chalcones (**3**) in good yields (Scheme 1). Compound **3i** was prepared from compound **3h** in the presence of (1-bromoethyl)benzene and K_2_CO_3_ in very good yield (92% yield, Scheme 1).

### 2.2. Cytotoxicity Levels of Chalcone-Derivative Compounds In Vitro

The solubility of the newly synthesized compounds **3f** and **3i** and the other chalcone derivatives was calculated theoretically by *c*log*P* (Table 1) [23]. The results showed that all the compounds, and in particular compound **3i**, presented a clear lipophilic behavior. Interestingly, all the compounds bore one to four hydrogen-bond acceptors, but only **3a** and **3h**, very promising in terms of activity, showed a hydrogen-bond acceptor, suggesting that this aspect could be a determinant for the anti-cancer effect on HGSOC cells (Table 1). Moreover, all the considered compounds are in accordance with Lipinski’s “rule of 5”, justifying them as potential drug candidates [24].

**Figure 1:**
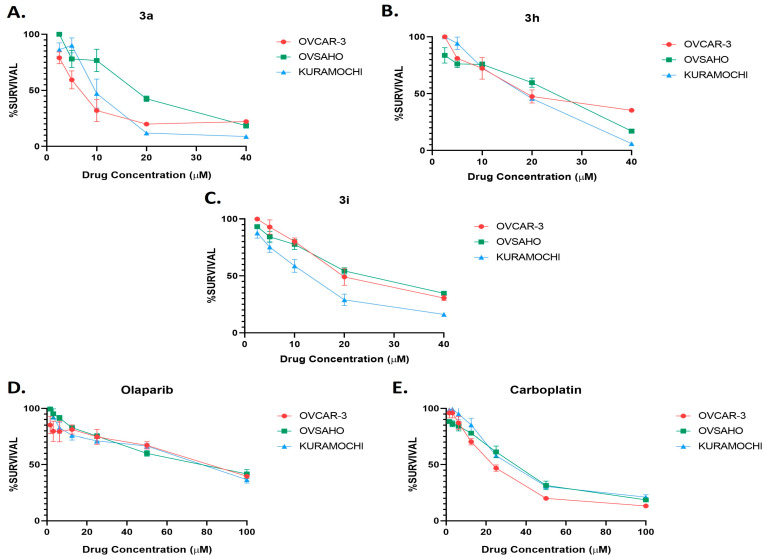
Cell viability analysis of compounds **3a** (**A**), **3h** (**B**), **3i** (**C**), Olaparib (**D**), and carboplatin (**E**) on OVCAR-3, OVSAHO, and KURAMOCHI cells. Cells were treated with increasing concentrations of the compounds (2.5–40 μM) for 72 h. Olaparib and carboplatin were used as reference drugs for HGSOC cells. All results were normalized to data of negative control DMSO.

The bioactivity of the small library of compounds was evaluated on high-grade serous ovarian cancer cell lines in vitro. A sulforhodamine B cell viability assay was performed to determine the half-maximal inhibitory concentration (IC_50_) value of the compounds. The compounds were dissolved in DMSO. OVCAR-3, OVSAHO, and KURAMOCHI cells were treated with increasing concentrations of the compounds (2.5–40 μM) and DMSO as a negative control for 72 h. Results were normalized to DMSO negative control measurements. The experiment was performed in triplicate. The representative survival graphs of the most active compounds are shown in Figure 1, and the rest of the compounds in Figure S1. The IC_50_ values of compounds indicated that they exhibited significant cytotoxic activity against high-grade serous ovarian cancer cells, as demonstrated in Table 1. Two reference drugs for ovarian cancer treatment were used to compare the activity of the reported chalcones. In particular, carboplatin is a widely used chemotherapy drug, and olaparib is a potent PARP-1 and PARP-2 inhibitor. Results showed that **3a**, **3h**, and **3i** had lower or similar IC_50_ values to carboplatin, whereas they presented significantly lower IC_50_ values than olaparib, indicating a higher activity on the ovarian cell lines used compared to the PARP inhibitor reference standard (Table 1 and Figure 1).

### 2.3. Cell Cycle Analysis by PI Staining

**Figure 2:**
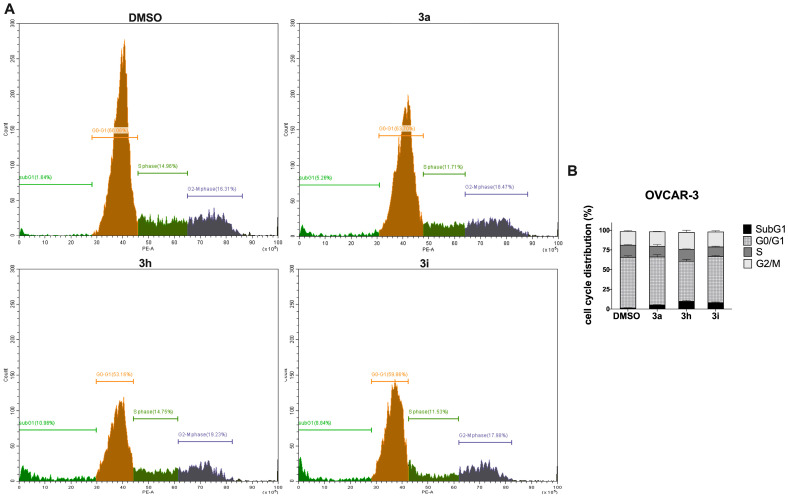
Cell cycle analysis of OVCAR-3 cells. (**A**) Representative cell cycle histograms after PI staining. Human high-grade serous ovarian cancer cell line, OVCAR-3, was treated with IC_75_ concentrations of chalcones **3a**, **3h**, and **3i** for 72 h, respectively, stained with PI and analyzed by flow cytometry. The first peak demonstrates G0/G1 phase (2N) (brown), the second peak demonstrates G2/M phase (4N) (purple), and in between represents S phase (green) cell populations. (**B**) The quantification of analysis is presented on bar graph. Error bars represent the SEM of data obtained in n = 2 independent experiments. OVCAR-3 cells treated with chalcones **3a**, **3h**, and **3i** showed a SubG1 increase compared to DMSO control.

To investigate the effects of the most active compounds **3a**, **3h**, and **3i** on the cell cycle, propidium iodide staining was performed. OVCAR-3 cells were either treated with IC_75_ concentrations of compounds or DMSO control for 72 h and stained with propidium iodide. The cell cycle distributions of OVCAR-3 cells after compound treatments are shown in Figure 2.

The results obtained from PI staining revealed that OVCAR-3 cells treated with compounds **3a**, **3h**, and **3i** separately showed a significant increase in SubG1 cell populations by changing the cell cycle characteristics of OVCAR-3 cells compared to the DMSO control. Since the increase in the SubG1 phase of the cell cycle correlates with the amount of fragmented DNA resulting from cell death, PI staining results suggested that chalcones **3a**, **3h**, and **3i** induced apoptotic cell death in OVCAR-3 cells, which required further investigation through apoptosis assay [25].

### 2.4. Chalcones ***3a***, ***3h***, and ***3i*** Induced Apoptotic Cell Death in OVCAR-3 Cells

**Figure 3:**
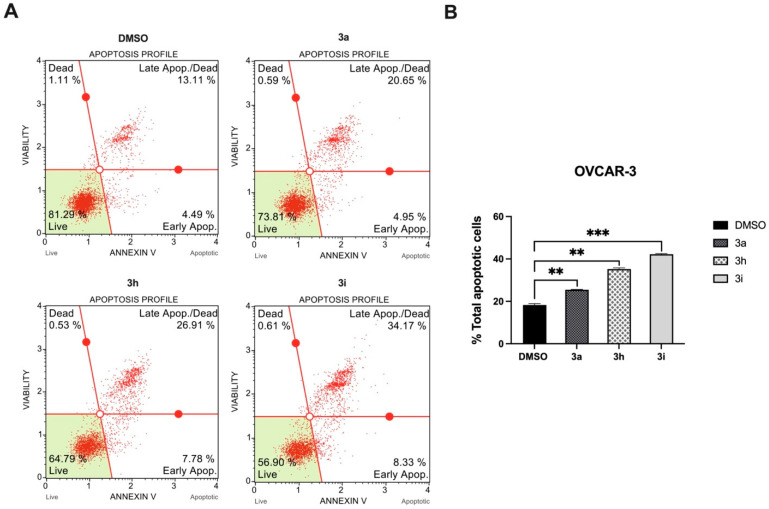
Apoptosis induction analysis of OVCAR-3 cells after 72 h compound treatment. (**A**) Representative plots of apoptosis. Human high-grade serous ovarian cancer cell line, OVCAR-3, was treated with IC_75_ concentrations of chalcones **3a**, **3h**, and **3i** for 72 h, respectively. (**B**) The quantification of analysis is presented on bar graph. Treatment with each chalcone resulted in a significant increase in %total apoptotic cell populations of OVCAR-3. Error bars represent the SEM of data obtained in *n* = 2 independent experiments. Statistical differences were analyzed with two-tailed Student’s *t*-test in comparison to DMSO ** *p* < 0.001, *** *p* < 0.0001.

To investigate the cell death mechanism induced by chalcones **3a**, **3h**, and **3i**, MUSE Annexin-V & Cell Death Assay was performed. After OVCAR-3 cells were treated with the compounds (IC_75_) for 72 h, the percentage of live, apoptotic, and dead cell populations was determined by the MUSE cell analyzer (Figure 3). The results revealed that OVCAR-3 cells treated with chalcones **3a**, **3h**, and **3i**, respectively, demonstrated a significant increase in total apoptotic cells compared to the DMSO control, as well as a prominent decrease in live cell populations. In particular, chalcone **3i** showed the most potent effect against OVCAR-3 cells, producing an increase in apoptotic cell death, while there was no significant change in Annexin-V (−) 7-AAD (+) cell populations (dead cells). The results obtained from MUSE Annexin-V & Cell Death Assay were compatible with the PI staining data showing a subG1 increase, which confirmed that chalcones **3a**, **3h**, and **3i** induced apoptotic cell death in OVCAR-3 cells.

## 3. Materials and Methods

### 3.1. General Information

All reactions were carried out in oven-dried glassware and dry solvents under nitrogen atmosphere. Unless otherwise stated, all solvents were purchased from Sigma-Aldrich and used without further purification. Substrates and reagents were purchased from Sigma-Aldrich and used as received. Thin-layer chromatography (TLC) was performed on Merck precoated 60F_254_ plates. Reactions were monitored by TLC on silica gel, with detection by UV light (254 nm). ^1^H NMR and ^13^C NMR spectra were recorded on a Bruker Avance NEO 400 MHz spectrometer (Ettlingen, Germany) at 400 MHz and 100 MHz, respectively. Chemical shifts are provided as *δ* values in ppm relative to residual solvent peaks as the internal reference. ^13^C NMR spectra are 1H-decoupled, and the determination of the multiplicities was achieved by the APT pulse sequence. HR-MS analyses were performed by using a Q-ToF SYNAPT G2-Si spectrometer with an electrospray ionization source (Waters, UK). All tested compounds possessed a purity of >98%, confirmed via elemental analyses (CHN) using a Perkin-Elmer CHN Analyzer Series II 2400 (Waltham, MA, USA). Melting points were determined on a Stuart Scientific melting point apparatus (SMP3) and are uncorrected, operating at a heating rate of 1 °C/min.

### 3.2. Synthetic Procedures

#### 3.2.1. General Synthetic Procedure for Title Compounds **3a**–**3h**

An aqueous solution of sodium hydroxide (30%, 25 mL) was slowly added to a methanol solution (30 mL) of the appropriate acetophenone (5.0 mmol). After the solution had been cooled to room temperature, the appropriate benzaldehyde (6.0 mmol) was added, and the solution was stirred overnight at the same temperature. After the completion of the reaction (12 h), the precipitated solid was removed by filtration, washed with H_2_O, and recrystallized from ethanol. If precipitation did not occur, the solvent was evaporated in vacuum. EtOAc was added, and the organic phase was washed with water and brine, dried with Na_2_SO_4_, filtered, and evaporated. The desired product was obtained after purification by flash column chromatography. For compounds **3d**, **3g**, and **3h**, the analytical characterization was in accordance with the literature.

##### **3a.** (*E*)-4-(3-(2-hydroxyphenyl)acryloyl)benzonitrile

According to the general procedure, the desired chalcone derivative **3a** was obtained from the reaction between 4-acetylbenzonitrile (5.0 mmol) and 2-hydroxybenzaldehyde (6.0 mmol) after purification by flash column chromatography (silica gel, eluent mixture 6:4 *n*-Hex/EtOAc) and evaporation of the solvent, as a yellow amorphous solid (55% yield). m.p. = 193–194 °C; ^1^H NMR (400 MHz, CDCl_3_): *δ* = 8.09 (2H, d, *J* = 8.4 Hz), 8.08 (1H, d, *J* = 16 Hz), 7.80 (2H, d, *J* = 8.4 Hz), 7.64 (1H, d, *J* = 16 Hz), 7.59 (1H, dd, *J* = 8.0 Hz *J* = 1.6 Hz), 7.31 (1H, td, *J* = 8.0 Hz *J* = 1.6 Hz), 7.00 (1H, t, *J* = 8.0 Hz), 6.85 (1H, d, *J* = 8.0 Hz). ^13^C NMR (100 MHz, CDCl_3_): *δ* = 190.0, 155.4, 142.0, 141.8, 132.4 (2C), 132.2, 130.2, 128.9 (2C), 122.4, 121.9, 121.4, 117.8, 116.5, 115.8; HRMS-ESI: *m*/*z* [M − H]^−^ calcd for C_16_H_10_NO_2_: 248.0712, found: 248.0710. Anal. Calcd for C_16_H_11_NO_2_: C, 77.10; H, 4.45; N, 5.62. Found: C, 77.32; H, 4.51, N, 5.56.

##### **3b**. (*E*)-4-(3-(4-(dimethylamino)phenyl)acryloyl)benzonitrile

According to the general procedure, the desired chalcone derivative **3b** was obtained from the reaction between 4-acetylbenzonitrile (5.0 mmol) and 4-(dimethylamino)benzaldehyde (6.0 mmol) after purification by flash column chromatography (silica gel, eluent mixture 65:35 *n*-Hex/EtOAc) and evaporation of the solvent, as a red amorphous solid (85% yield). m.p. = 166–167 °C; ^1^H NMR (400 MHz, CDCl_3_): *δ* = 8.06 (2H, d, *J* = 8.4 Hz), 7.81 (1H, d, *J* = 15.6 Hz), 7.78 (2H, d, *J* = 8.4 Hz), 7.56 (2H, d, *J* = 8.4 Hz), 7.26 (1H, d, *J* = 15.6 Hz), 6.70 (2H, d, *J* = 8.4 Hz), 3.07 (6H, s). ^13^C NMR (100 MHz, CDCl_3_): *δ* = 189.0, 152.5, 147.6, 142.6, 132.3 (2C), 130.9 (2C), 128.7 (2C), 122.1, 118.3, 115.8, 115.2, 111.8 (2C), 40.09 (2C); HRMS-ESI: *m*/*z* [M + H]^+^ calcd for C_18_H_17_N_2_O: 277.1341, found: 277.1345. Anal. Calcd for C_18_H_16_N_2_O: C, 78.24; H, 5.84; N, 10.14. Found: C, 78.47; H, 5.91, N, 10.07.

##### **3c**. (*E*)-4-(3-(benzo[d][1,3]dioxol-5-yl)acryloyl)benzonitrile

According to the general procedure, the desired chalcone derivative **3c** was obtained from the reaction between 4-acetylbenzonitrile (5.0 mmol) and piperonal (6.0 mmol) after precipitation of the product, filtration, and recrystallization from ethanol, as a yellow amorphous solid (85% yield). m.p. = 182–183 °C; ^1^H NMR (400 MHz, DMSO-*d*_6_): *δ* = 8.27 (2H, d, *J* = 8.4 Hz), 8.03 (2H, d, *J* = 8.4 Hz), 7.81 (1H, d, *J* = 15.6 Hz), 7.71 (1H, d, *J* = 15.6 Hz), 7.66 (1H, d, *J* = 1.6 Hz), 7.34 (1H, dd, *J* = 8.0 Hz *J* = 1.6 Hz), 6.99 (1H, d, *J* = 8.0 Hz), 6.12 (2H, s). ^13^C NMR (100 MHz, DMSO-*d*_6_): *δ* = 188.6, 150.4, 148.6, 145.9, 141.5, 133.2 (2C), 129.5 (2C), 127.0, 120.0, 118.7 (2C), 115.4, 109.0, 107.5, 102.2; HRMS-ESI: *m*/*z* [M + Na]^+^ calcd for C_17_H_11_NO_3_Na: 300.0637, found: 300.0635. Anal. Calcd for C_17_H_11_NO_3_: C, 73.64; H, 4.00; N, 5.05. Found: C, 73.85; H, 4.07, N, 4.97.

##### **3d**. (*E*)-3-(4-(pyrrolidin-1-yl)phenyl)-1-(p-tolyl)prop-2-en-1-one [26]

According to the general procedure, the desired chalcone derivative **3d** was obtained from the reaction between 4′-methylacetophenone (5.0 mmol) and 4-(1-pyrrolidino)benzaldehyde (6.0 mmol) after purification by flash column chromatography (silica gel, eluent mixture 6:4 *n*-Hex/EtOAc) and evaporation of the solvent, as a light brown amorphous solid (80% yield). m.p. = 180–181 °C; HRMS-ESI: *m*/*z* [M + Na]^+^ calcd for C_20_H_21_NONa: 314.1521, found: 314.1522. Anal. Calcd for C_20_H_21_NO: C, 82.44; H, 7.26; N, 4.81. Found: C, 82.69; H, 7.33, N, 4.74.

##### **3e**. (*E*)-3-(benzo[d][1,3]dioxol-5-yl)-1-(p-tolyl)prop-2-en-1-one

According to the general procedure, the desired chalcone derivative **3e** was obtained from the reaction between 4′-methylacetophenone (5.0 mmol) and piperonal (6.0 mmol) after precipitation of the product, filtration, and recrystallization from ethanol, as a yellow amorphous solid (85% yield). m.p. = 132–133 °C; ^1^H NMR (400 MHz, DMSO-*d*_6_): *δ* = 8.07 (2H, d, *J* = 8.4 Hz), 7.81 (1H, d, *J* = 15.6 Hz), 7.67 (1H, d, *J* = 15.6 Hz), 7.65 (1H, d, *J* = 1.2 Hz), 7.36 (2H, d, *J* = 8.0 Hz), 7.32 (1H, dd, *J* = 8.4 Hz *J* = 1.2 Hz), 6.98 (1H, d, *J* = 8.0 Hz), 6.11 (2H, s), 2.40 (3H, s). ^13^C NMR (100 MHz, DMSO-*d*_6_): *δ* = 188.9, 150.0, 148.6, 144.1, 143.8, 135.7 (2C), 129.8 (2C), 129.1 (2C), 126.3, 120.5, 109.0, 107.4, 102.1, 21.63; HRMS-ESI: *m*/*z* [M + Na]^+^ calcd for C_17_H_14_ONa: 289.0841, found: 289.0842. Anal. Calcd for C_17_H_14_O_3_: C, 76.68; H, 5.30. Found: C, 76.91; H, 5.36.

##### **3f**. (*E*)-4-(3-(4-(benzyloxy)phenyl)acryloyl)benzonitrile

According to the general procedure, the desired chalcone derivative **3f** was obtained from the reaction between 4-acetylbenzonitrile (5.0 mmol) and 4-(benzyloxy)benzaldehyde (6.0 mmol) after precipitation of the product, filtration, and recrystallization from ethanol, as a yellow amorphous solid (85% yield). m.p. = 153–154 °C; ^1^H NMR (400 MHz, DMSO-*d*_6_): *δ* = 8.27 (2H, d, *J* = 8.4 Hz), 8.04 (2H, d, *J* = 8.4 Hz), 7.88 (2H, d, *J* = 8.8 Hz), 7.82 (1H, d, *J* = 15.2 Hz), 7.76 (1H, d, *J* = 15.6 Hz), 7.48–7.32 (5H, m), 7.11 (2H, d, *J* = 8.8 Hz), 5.19 (2H, s). ^13^C NMR (100 MHz, DMSO-*d*_6_): *δ* = 188.7, 161.2, 145.8, 141.6, 137.1, 133.2 (2C), 131.6 (2C), 129.5 (2C), 128.9 (2C), 128.4, 128.2 (2C), 127.8, 119.7, 118.7, 115.8 (2C), 115.3, 69.92; HRMS-ESI: *m*/*z* [M + Na]^+^ calcd for C_23_H_17_NO_2_Na: 362.1157, found: 362.1160. Anal. Calcd for C_23_H_17_NO_2_: C, 81.40; H, 5.05; N, 4.13. Found: C, 81.65; H, 5.12, N, 4.17.

##### **3g**. (*E*)-3-(4-(dimethylamino)phenyl)-1-phenylprop-2-en-1-one [27]

According to the general procedure, the desired chalcone derivative **3g** was obtained from the reaction between acetophenone (5.0 mmol) and 4-(dimethylamino)benzaldehyde (6.0 mmol) after purification by flash column chromatography (silica gel, eluent mixture 7:3 *n*-Hex/EtOAc) and evaporation of the solvent, as a dark yellow amorphous solid (75% yield). m.p. = 106–107 °C; HRMS-ESI: *m*/*z* [M + Na]^+^ calcd for C_17_H_17_NONa: 274.1208, found: 274.1205. Anal. Calcd for C_17_H_17_NO: C, 81.24; H, 6.82; N, 5.57. Found: C, 81.49; H, 6.88, N, 5.51.

##### **3h**. (*E*)-3-(4-hydroxyphenyl)-1-(p-tolyl)prop-2-en-1-one [28]

According to the general procedure, the desired chalcone derivative **3h** was obtained from the reaction between 4′-methylacetophenone (5.0 mmol) and 4-hydroxybenzaldehyde (6.0 mmol) after purification by flash column chromatography (silica gel, eluent mixture 5:5 *n*-Hex/EtOAc) and evaporation of the solvent, as yellow crystals (65% yield). m.p. = 161–162 °C; HRMS-ESI: *m*/*z* [M − H]^−^ calcd for C_16_H_13_O_2_: 237.0916, found: 237.0915. Anal. Calcd for C_16_H_14_O_2_: C, 80.65; H, 5.92. Found: C, 80.89; H, 6.01.

#### 3.2.2. Synthesis of (E)-3-(4-(1-phenylethoxy)phenyl)-1-(p-tolyl)prop-2-en-1-one (3i)

K_2_CO_3_ (0.145 g, 1.05 mmol) and (1-bromoethyl)benzene (0.035 mL, 0.25 mmol) were added to a solution of compound **3h** (0.050 g, 0.21 mmol) in acetone (0.73 mL) and the reaction mixture was stirred at reflux until completion (3 h). Then, water was added, and the aqueous phase was extracted with EtOAc. The organic phase was dried with Na_2_SO_4_, filtered, and evaporated to provide compound **3i** after purification by flash column chromatography (silica gel, eluent mixture 7:3 *n*-Hex/EtOAc) and evaporation of the solvent as light brown solid (92% yield). m.p. = 88–89 °C; ^1^H NMR (400 MHz, DMSO-*d*_6_): *δ* = 8.03 (2H, d, *J* = 8.4 Hz), 7.75 (2H, d, *J* = 8.8 Hz), 7.74 (1H, d, *J* = 15.6 Hz), 7.64 (1H, d, *J* = 15.6 Hz), 7.43 (2H, d, *J* = 8.4 Hz), 7.37–7.24 (5H, m), 6.98 (2H, d, *J* = 8.8 Hz), 5.61 (1H, q, *J* = 6.4 Hz), 2.39 (3H, s), 1.58 (3H, d, *J* = 6.4 Hz). ^13^C NMR (100 MHz, DMSO-*d*_6_): *δ* = 188.9, 160.0, 144.0, 143.8, 143.1, 135.8, 131.0 (2C), 129.8 (2C), 129.0 (4C), 128.0, 127.8, 126.2 (2C), 120.1, 116.5 (2C), 75.41, 24.49, 21.62; HRMS-ESI: *m*/*z* [M + Na]^+^ calcd for C_24_H_22_O_2_Na: 365.1517, found: 365.1520. Anal. Calcd for C_24_H_22_O_2_: C, 84.18; H, 6.48. Found: C, 84.43; H, 6.53.

### 3.3. Cell Culture

All cell lines were cultured in RPMI-1640 with L-glutamine (Capricorn, Düsseldorf, Germany) supplemented with 10% FBS (Biowest, Nuaillé, France) and 1% Penicillin/Streptomycin (Biowest, Nuaillé, France). Cells were cultured in tissue culture flasks, maintained at 37 °C in a humidified incubator with 5% CO_2_, and routinely checked for *Mycoplasma* contamination. KURAMOCHI (JCRB0098) and OVSAHO (JCRB1046) cell lines were purchased from the Japanese Collection of Research Bioresources (JCRB) Cell Bank (Osaka, Japan). OVCAR3 (HTB-161) cells were purchased from ATCC (Manassas, VA, USA).

### 3.4. NCI-Sulforhodamine B (SRB) Cell Viability Assay

Cells were seeded into 96-well plates at 5000 cells/well and allowed to attach for 24 h. On day 1, triplicate wells were treated with five serial dilutions (1:2) of compounds (0−40 mM). After 72 h incubation, cells were fixed with 10% (*v*/*v)* ice-cold trichloroacetic acid (Sigma-Aldrich, St. Louis, MO, USA) for 1 h at 4 °C. After washing cells four times with distilled water and leaving to air-dry, cells were stained with 0.4% (*w*/*v*) SRB (Sigma-Aldrich, USA) in 1% (*v*/*v*) acetic acid (Isolab, Germany) at RT, dark for 30 min. The plates were washed five times with 1% (*v*/*v*) acetic acid and left to air-dry. The SRB stain was dissolved in 10 mM Tris-base solution (Biorad, Hercules, CA, USA), and absorbance was measured at 564 nm in a microplate reader (Synergy H1 Plate Reader, Biotek, Winooski, VT, USA). The data were presented as IC_50_, normalized to DMSO-treated samples as a negative control. The experiment was performed in triplicate.

### 3.5. Cell Cycle Analysis with PI Staining

Cells were seeded into 6-well plates at 1 × 10^5^ cells/well density and incubated for 24 h. Cells were treated with an IC_75_ concentration of each compound or DMSO control. After 72 h incubation, cells were trypsinized and collected into 15 mL falcon tubes (spent medium included). Samples were centrifuged at 2000 rpm for 6 min. The supernatant was discarded, and the pellet was resuspended in ice-cold 1 × DPBS. Samples were centrifuged at 2000 rpm for 6 min again, and the cell pellet was dissolved in 1 mL 1 × DPBS. While vortexing, 2.5 mL ice-cold absolute ethanol (70% final percentage) was slowly added to the tubes for fixation. Samples were kept at 4 °C for up to one week. For staining protocol, the fixed samples were centrifuged at 1500 rpm for 5 min at 4 °C. Supernatant was removed, and the cell pellet was resuspended in propidium iodide (PI) solution, followed by a 40 min incubation at 37 °C, dark. 1 × DPBS was added to the cells, and samples were again centrifuged at 1500 rpm for 5 min at 4 °C. Lastly, the cell pellet was resuspended in 1 × DPBS and transferred to the Beckman Coulter CytoFLEX Flow Cytometer (Beckman Coulter, Brea, CA, USA) for running 10,000 events per sample. Data were analyzed using CytExpert software version 2.5, and gates were determined according to DMSO control. The experiment was performed in duplicate.

### 3.6. Annexin-V/7-AAD Apoptosis Assay

OVCAR-3 cells were harvested from 6-well plates (1 × 10^5^ cells/well) following the corresponding treatment with compounds (IC_75_—72h), and apoptosis was assessed by using the Muse^®^ Annexin V & Dead Cell Kit (Luminex, Austin, TX, USA) according to the manufacturer’s instructions. The experiment was performed in duplicate.

## 4. Conclusions

In this study, a small library of chalcone derivatives, bearing electron-withdrawing or electron-donating substituents on both aromatic rings, was tested against high-grade serous ovarian cancer cells in vitro and compared with two known ovarian cancer cell drugs, carboplatin, and olaparib. Our results showed that three of them, **3a**, **3h**, and **3i**, presented high cytotoxic and anti-proliferative effects against OVCAR-3, OVSAHO, and KURAMOCHI cell lines, with IC_50_ concentrations < 25 µM, lower, in most of the cases, or similar to the reference drugs. To clarify the molecular mechanisms of these chalcones, we studied the cell cycle characteristics of HGSOC cells, showing a significant increase in SubG1 cell population and resulting in subG1 cell cycle arrest, which suggested apoptosis induction. To prove this finding, we also performed an apoptosis assay through the detection of the translocation of phosphatidylserine to the outer surface of the cell membrane, which is one of the characteristics of apoptosis, and we showed that the three chalcones caused a significant increase in apoptotic OVCAR-3 cell populations. Remarkably, among them, chalcone **3i** showed the highest effect on apoptosis-induced cell death mechanism.

This is the first study to show the cytotoxic effects of the chalcone derivatives **3a**, **3h**, and **3i** on ovarian cancer, and it considers them promising candidates to improve ovarian cancer treatment. However, further studies are needed to elucidate the molecular pathways of these synthesized chalcones on ovarian cancer.

## Figures and Tables

**Table 1:** IC_50_ values and calculated physicochemical properties of chalcone derivatives on high-grade serous ovarian cancer cells in vitro.

Compound	IC_50_ (µM) ^a^			Physicochemical Properties		
	OVCAR-3	OVSAHO	KURAMOCHI	*c*log*P* ^c^	n HBD ^d^	n HBA ^e^
**3a**	7.1 ± 1.7	16.2 ± 0.9	9.7 ± 0.7	3.3	1	3
**3b**	NI ^b^	NI ^b^	39.2 ± 28.8	3.7	0	2
**3c**	33.5 ± 4.1	NI ^b^	NI ^b^	3.5	0	4
**3d**	NI ^b^	NI ^b^	15.7 ± 0.2	4.8	0	1
**3e**	13.8 ± 1.5	27.3 ± 3.8	NI ^b^	4.2	0	3
**3f**	NI ^b^	NI ^b^	NI ^b^	5.2	0	3
**3g**	14.4 ± 1.3	38.4 ± 2.8	13.8 ± 0.6	3.9	0	1
**3h**	20.8 ± 3.1	17.8 ± 1.6	15.1 ± 0.7	3.8	1	2
**3i**	21.7 ± 2.4	24.3 ± 1.8	11.3 ± 1.4	6.3	0	2
Carboplatin	20.9 ± 2.2	27.7 ± 0.9	32 ± 1.3			
Olaparib	75.8 ± 6.1	97.3 ± 1.7	84.5 ±11.4			

^a^ Values are the mean ± SD of three independent experiments. ^b^ No Inhibition. ^c^ *c*log*P* = calculated octanol–water partition coefficient. ^d^ n HBD = number of hydrogen-bond donors. ^e^ n HBA = number of hydrogen-bond acceptors.

## Data Availability

Data are contained within the article.

## Supplementary Materials

The following supporting information can be downloaded at: https://www.mdpi.com/article/10.3390/molecules28237777/s1, Figure S1: Cell viability analysis of compounds **3b**(**A**), **3c**(**B**), **3d**(**C**), **3e**(**D**), **3f**(**E**) and **3g**(**F**) on OVCAR-3, OVSAHO, and KURAMOCHI cells. Cells were treated with increasing concentrations of the compounds (2.5–40 μM) for 72 h. All results were normalized to data of negative control DMSO.
