# Supplementary material for: Targeting Ovarian Cancer with Chalcone Derivatives: Cytotoxicity and Apoptosis Induction in HGSOC Cells
Source: Molecules. 2023 Nov 25;28(23):7777. doi: 10.3390/molecules28237777 (PMC10708092; doi:10.3390/molecules28237777)
Supplement: Supplementary file 1 [file molecules-28-07777-s001.zip › molecules-2696815-supplementary.pdf]

Supplementary Materials

Targeting Ovarian Cancer with Chalcone Derivatives: Cytotoxicity and Apoptosis Induction in HGSOC Cells

Elif Merve Aydin[1,†], İdil Su Canıtez[1,†], Eleonora Colombo[2,3], Salvatore Princiotto[4], Daniele Passarella[2], Sabrina Dallavalle[4], Michael S. Christodoulou[4,*] and Irem Durmaz Şahin[5,*]

[1]Koç University Research Center for Translational Medicine (KUTTAM), Sariyer, Istanbul, 34450, Turkey

[2]Dipartimento di Chimica, Università degli Studi di Milano, 20133 Milano, Italy

[3]Ann Romney Center for Neurologic Diseases, Department of Neurology, Brigham and Women's Hospital and Harvard Medical School, Boston, MA 02115, USA

[4]Department of Food, Environmental and Nutritional Sciences (DeFENS), University of Milan, via Celoria 2, 20133 Milan, Italy

[5]Koç University, School of Medicine, Sariyer, Istanbul, 34450, Turkey

†These authors contributed equally to this work.

***Correspondence:** irsahin@ku.edu.tr, michail.christodoulou@unimi.it

Table of contents

NMR spectra of compounds **3a – 3c, 3e, 3f, 3i** S2

Supplementary Figure S1 S17

3a. (*E*)-4-(3-(2-hydroxyphenyl)acryloyl)benzonitrile.

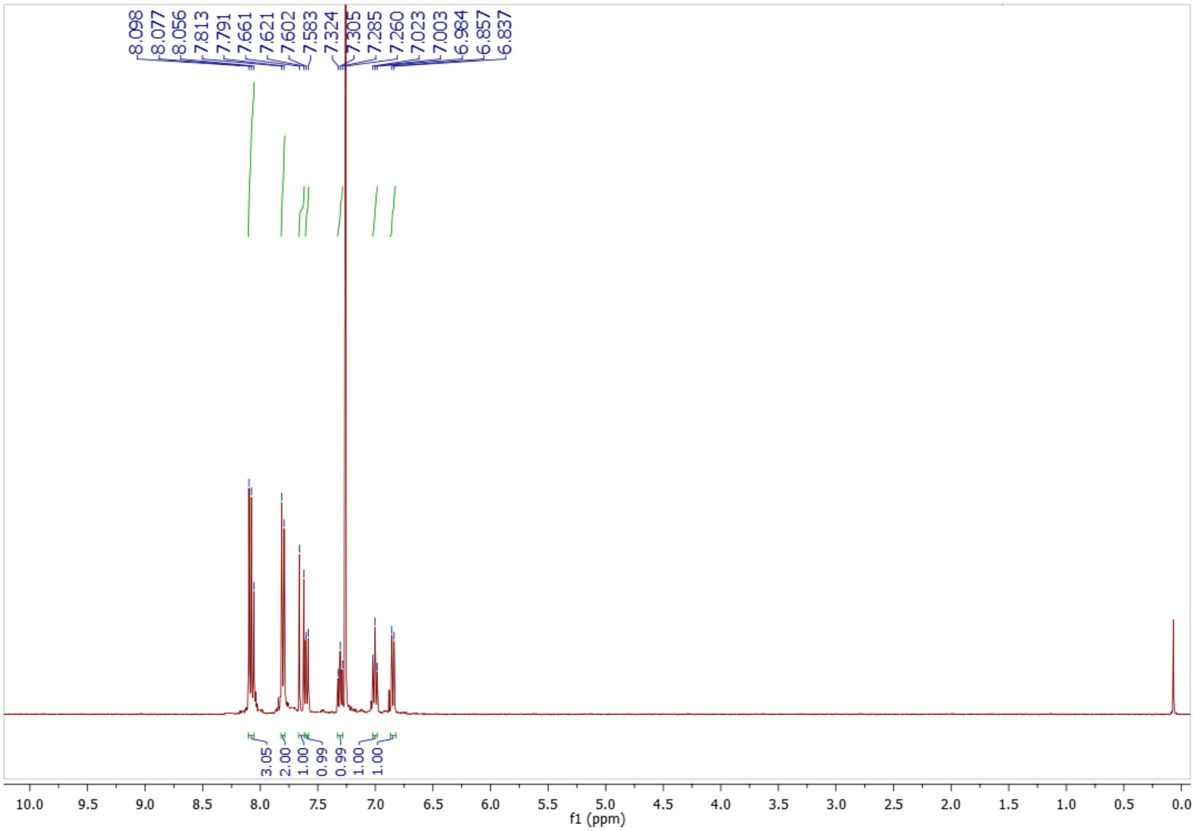

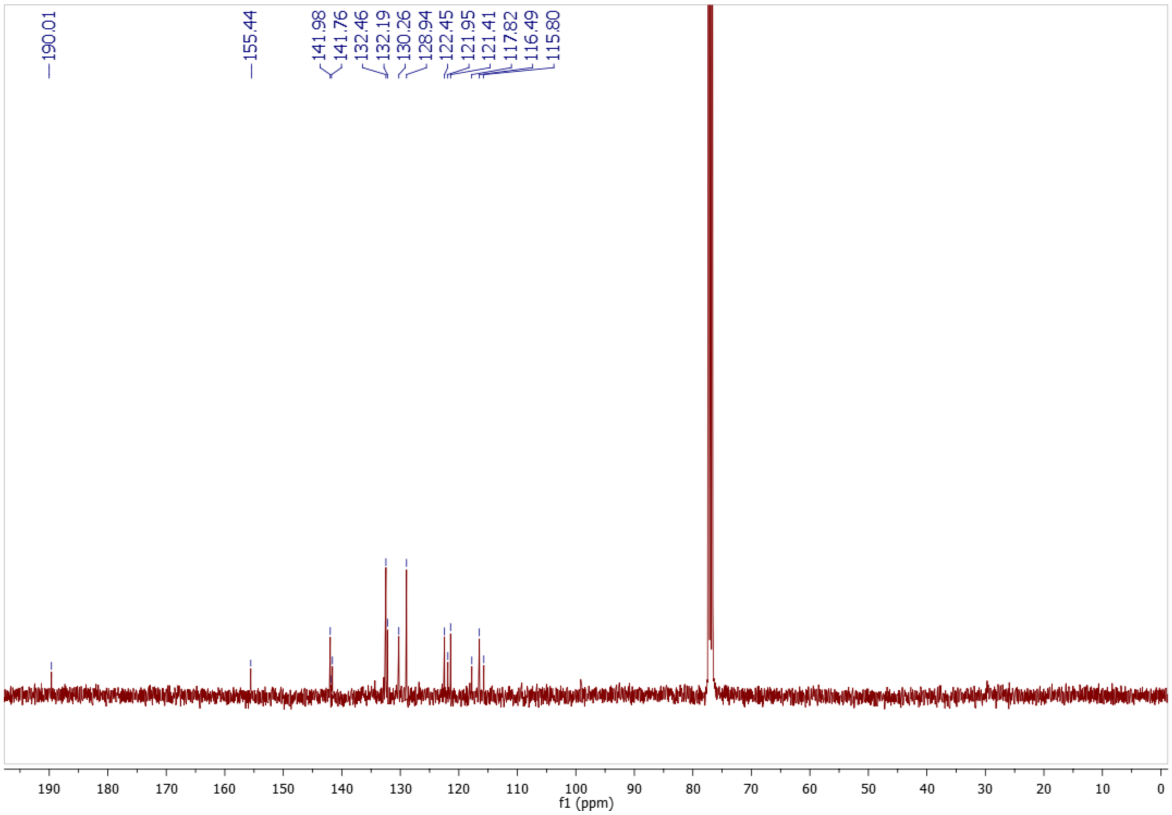

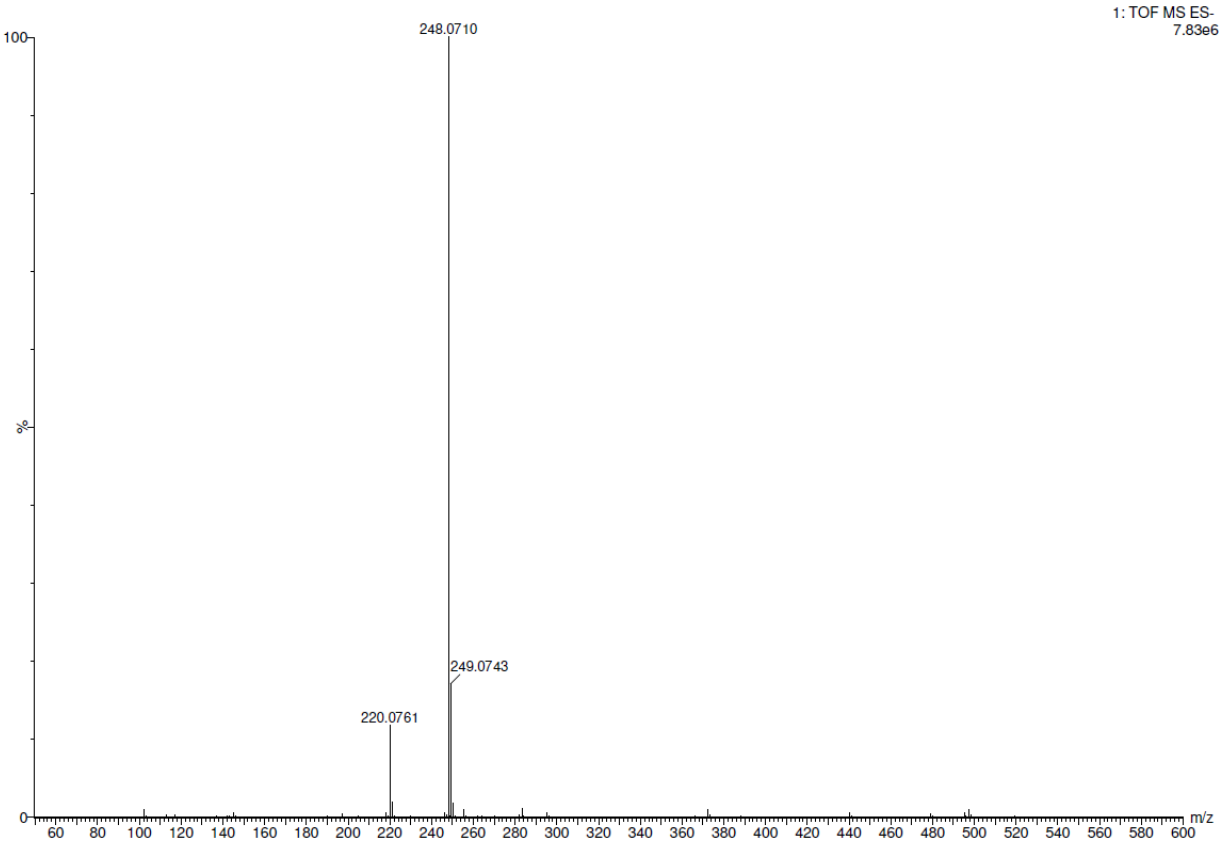
1: TOF MS ES-
7.83e6
248.0710
249.0743
220.0761
100
%
0
m/z
60
80
100
120
140
160
180
200
220
240
260
280
300
320
340
360
380
400
420
440
460
480
500
520
540
560
580
600

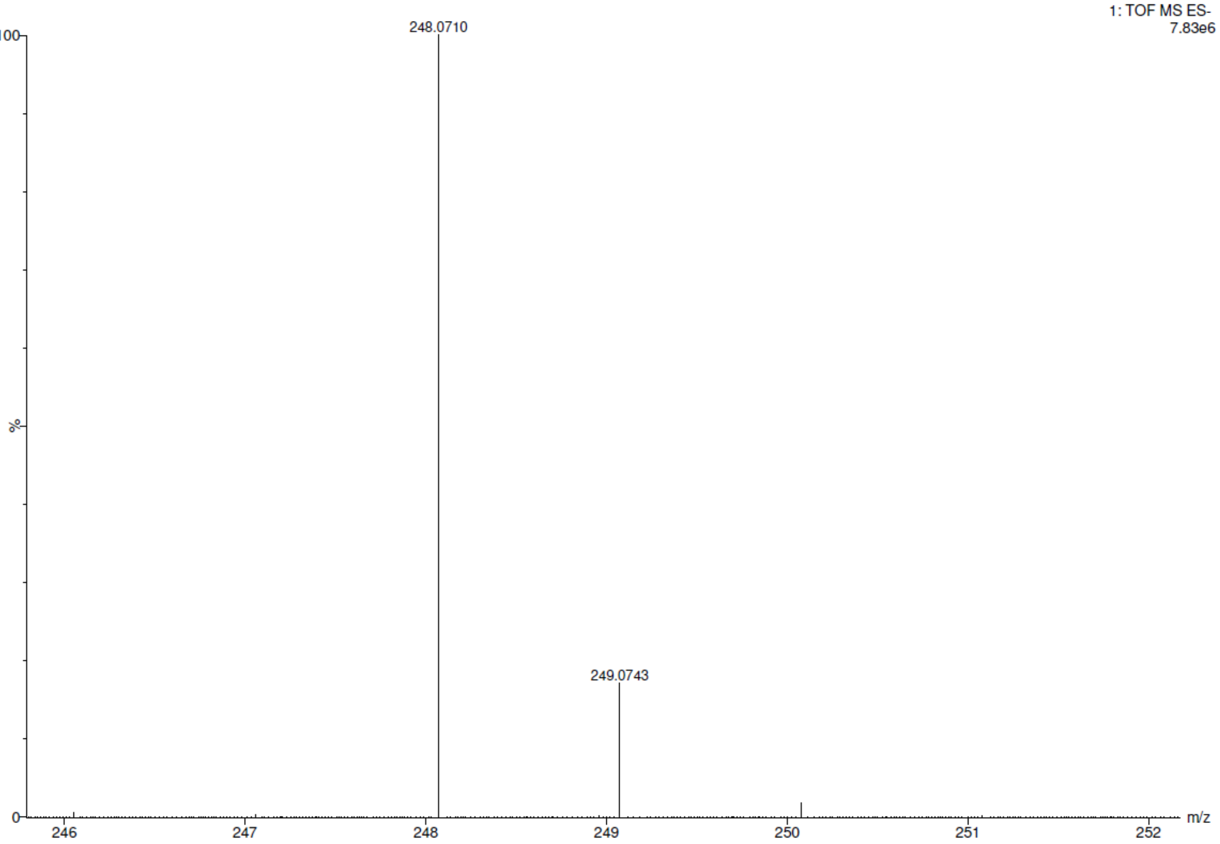
1: TOF MS ES-
7.83e6
248.0710
249.0743
100
%
0
m/z
246
247
248
249
250
251
252

3b. (*E*)-4-(3-(4-(dimethylamino)phenyl)acryloyl)benzonitrile.

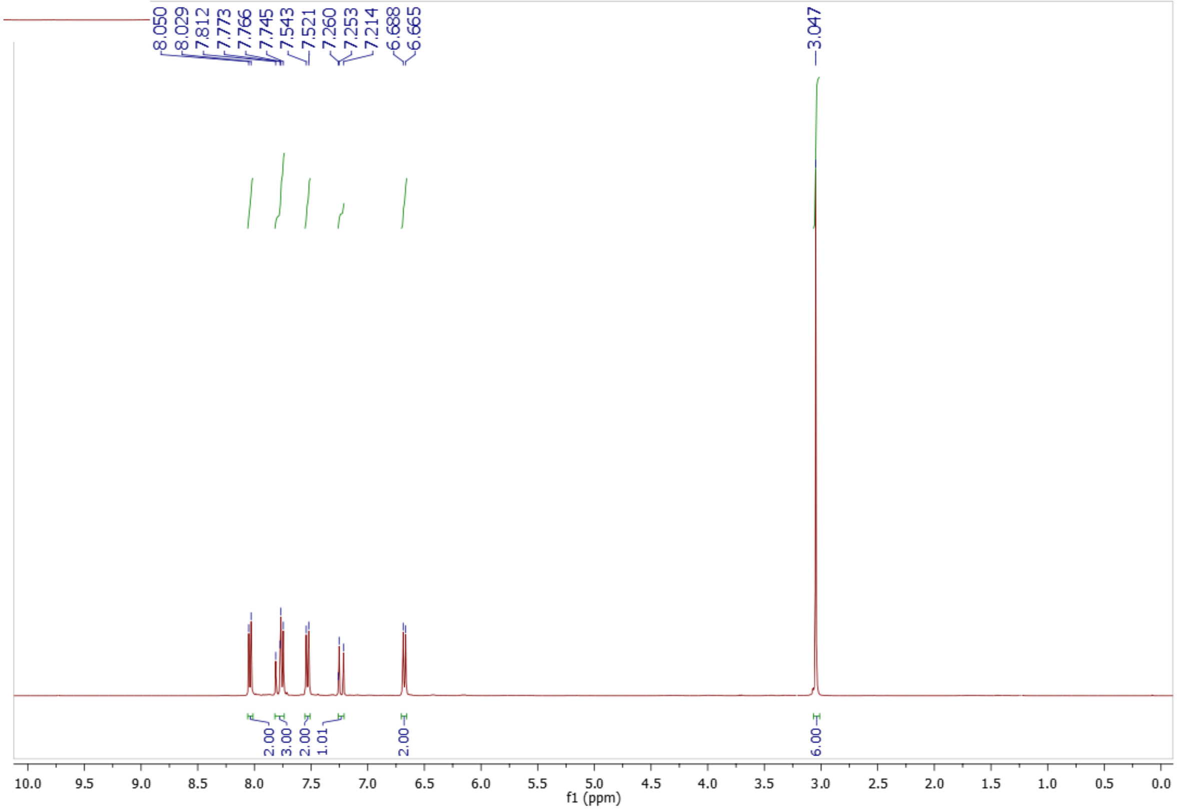

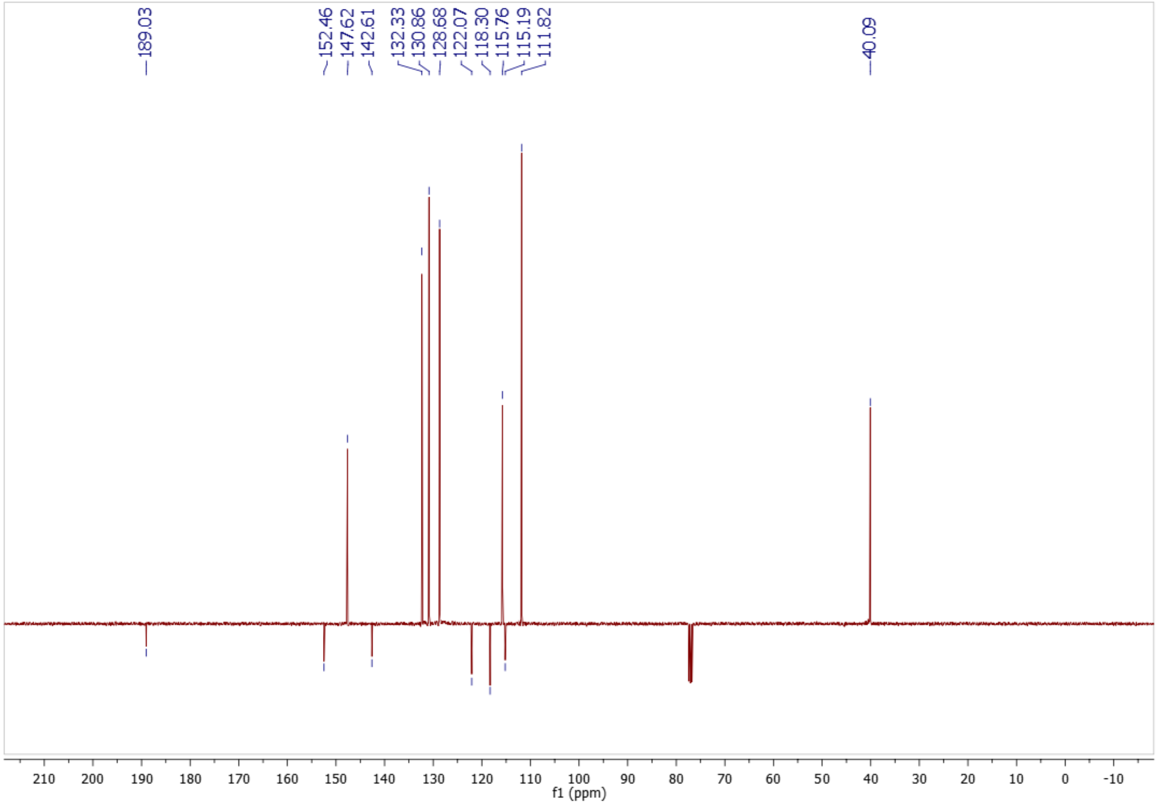

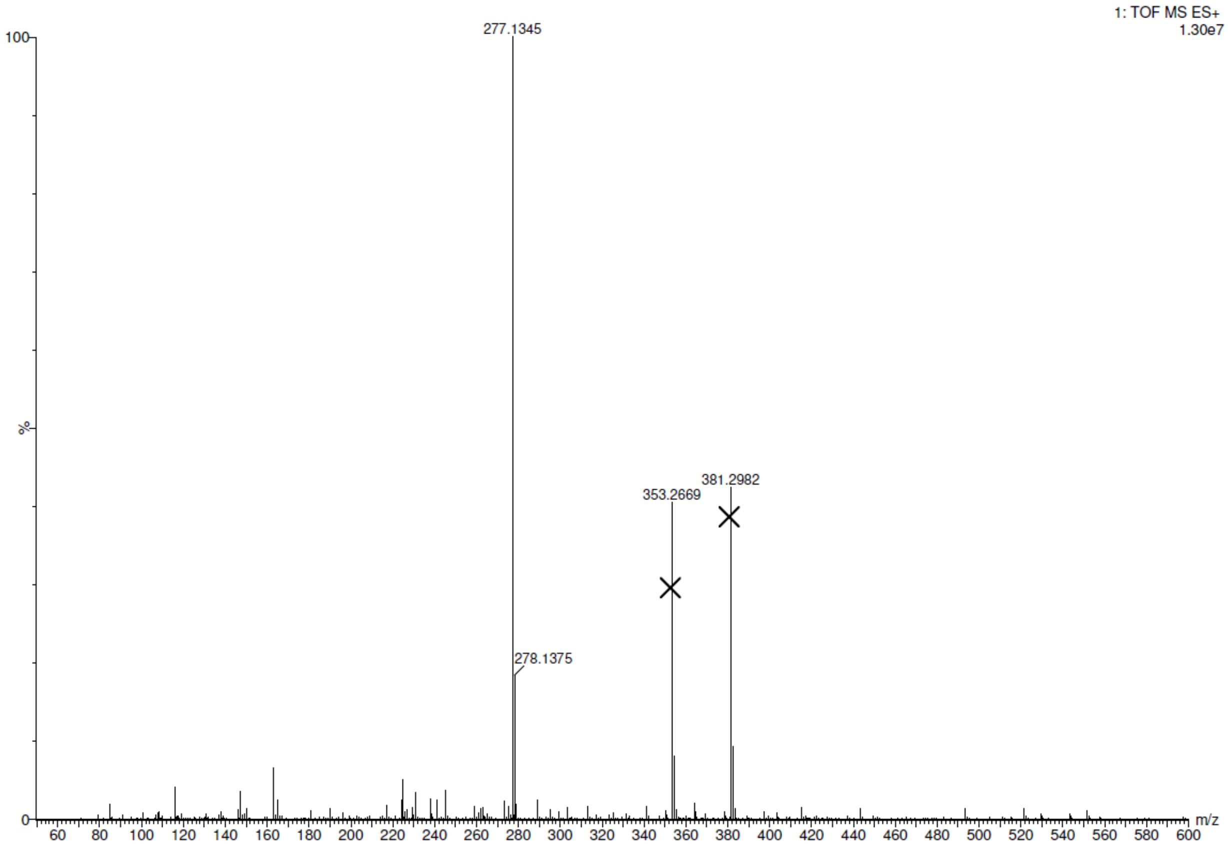
1: TOF MS ES+
1.30e7
277.1345
278.1375
353.2669
381.2982
%
m/z

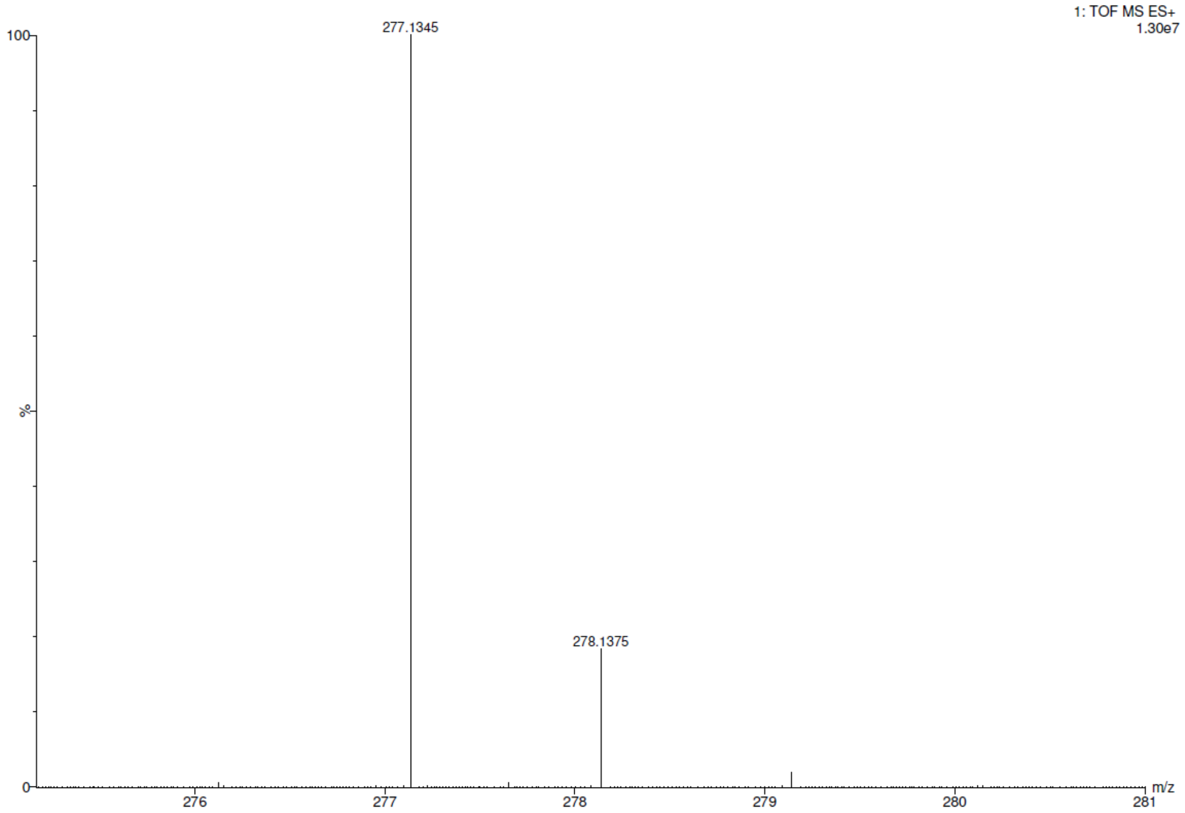
1: TOF MS ES+
1.30e7
277.1345
278.1375
%
m/z

3c. (*E*)-4-(3-(benzo[*d*][1,3]dioxol-5-yl)acryloyl)benzonitrile.

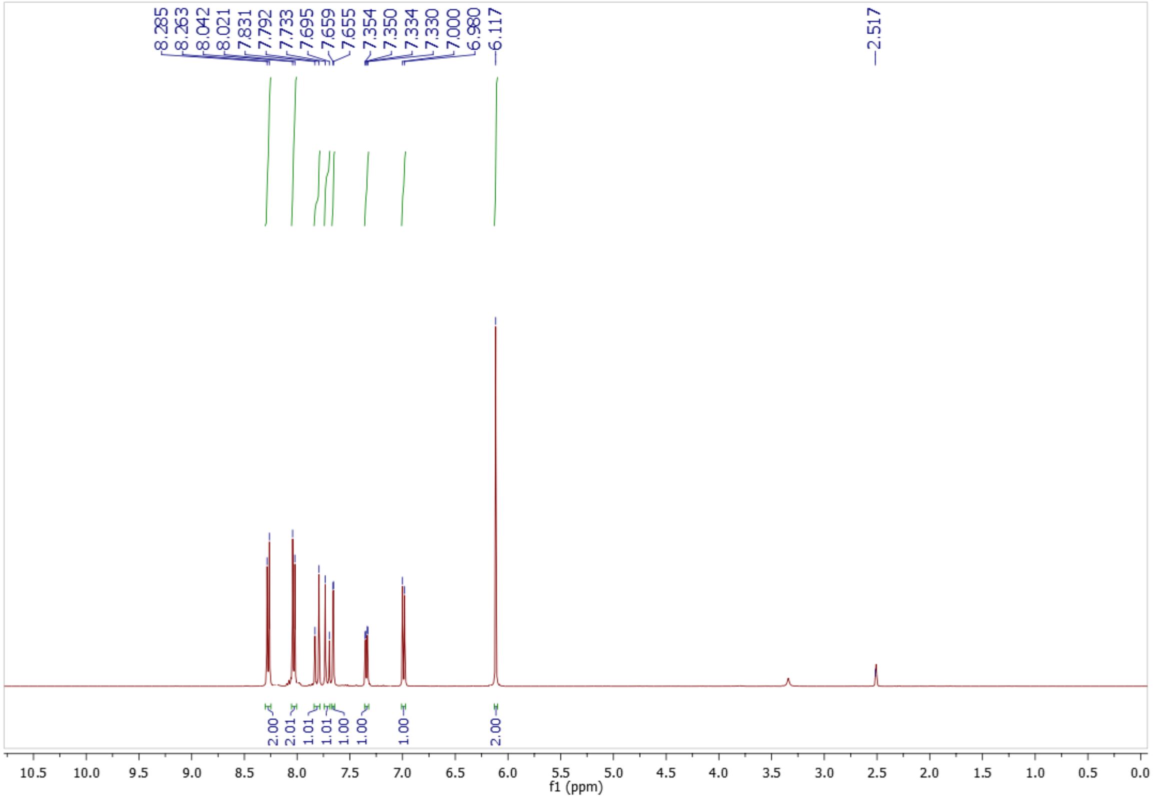

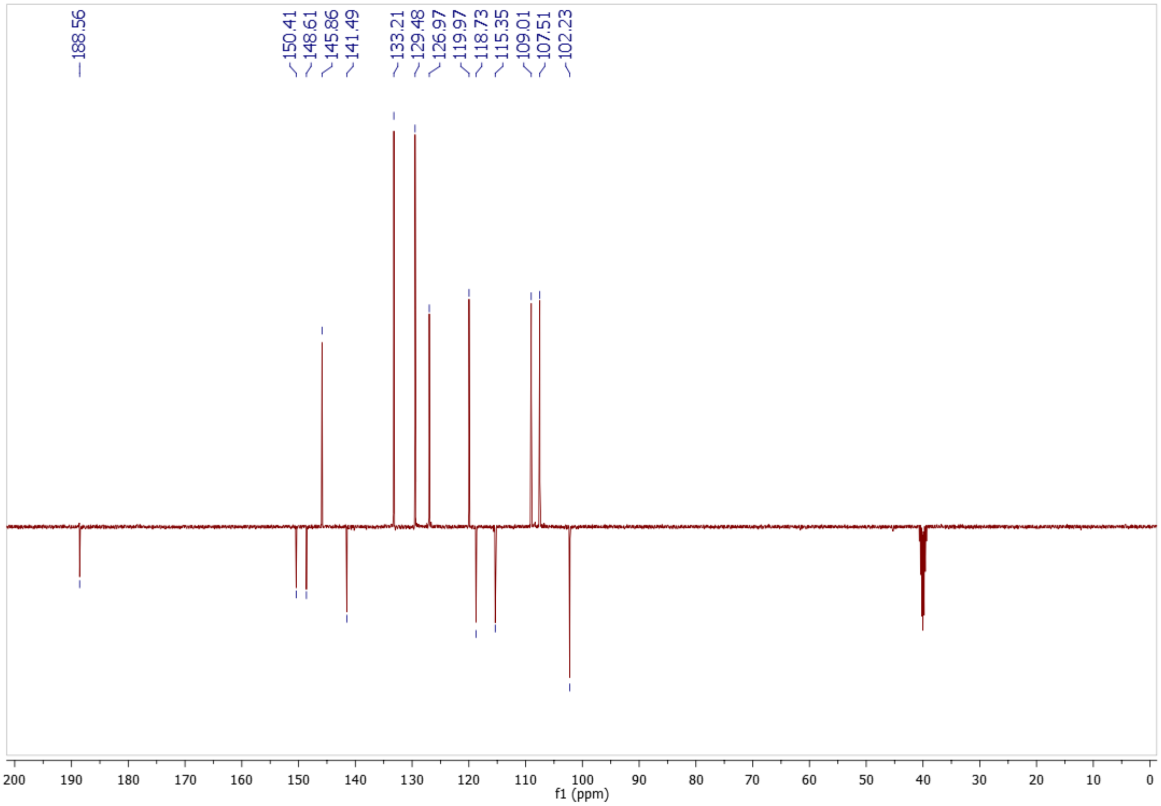

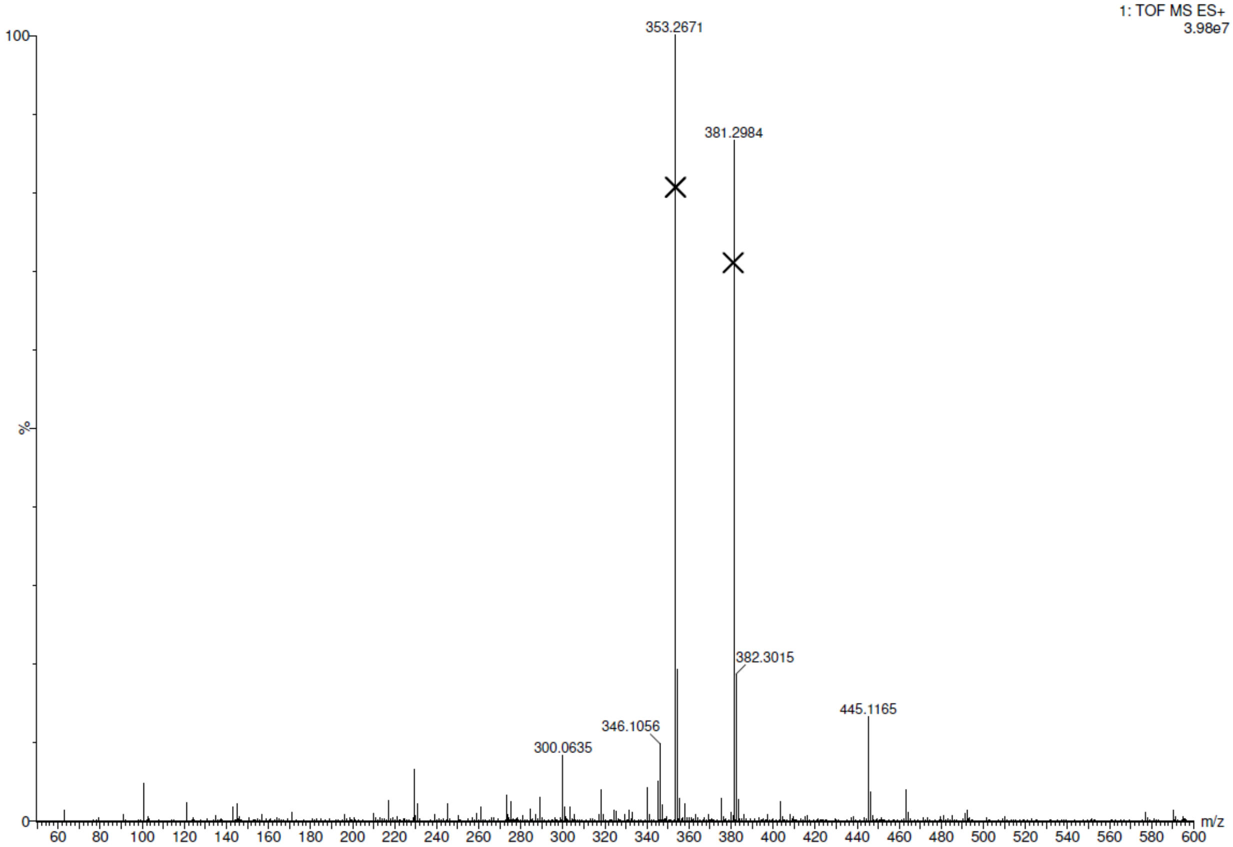
1: TOF MS ES+
3.98e7
353.2671
381.2984
382.3015
445.1165
346.1056
300.0635
100
%
0
60
80
100
120
140
160
180
200
220
240
260
280
300
320
340
360
380
400
420
440
460
480
500
520
540
560
580
600
m/z

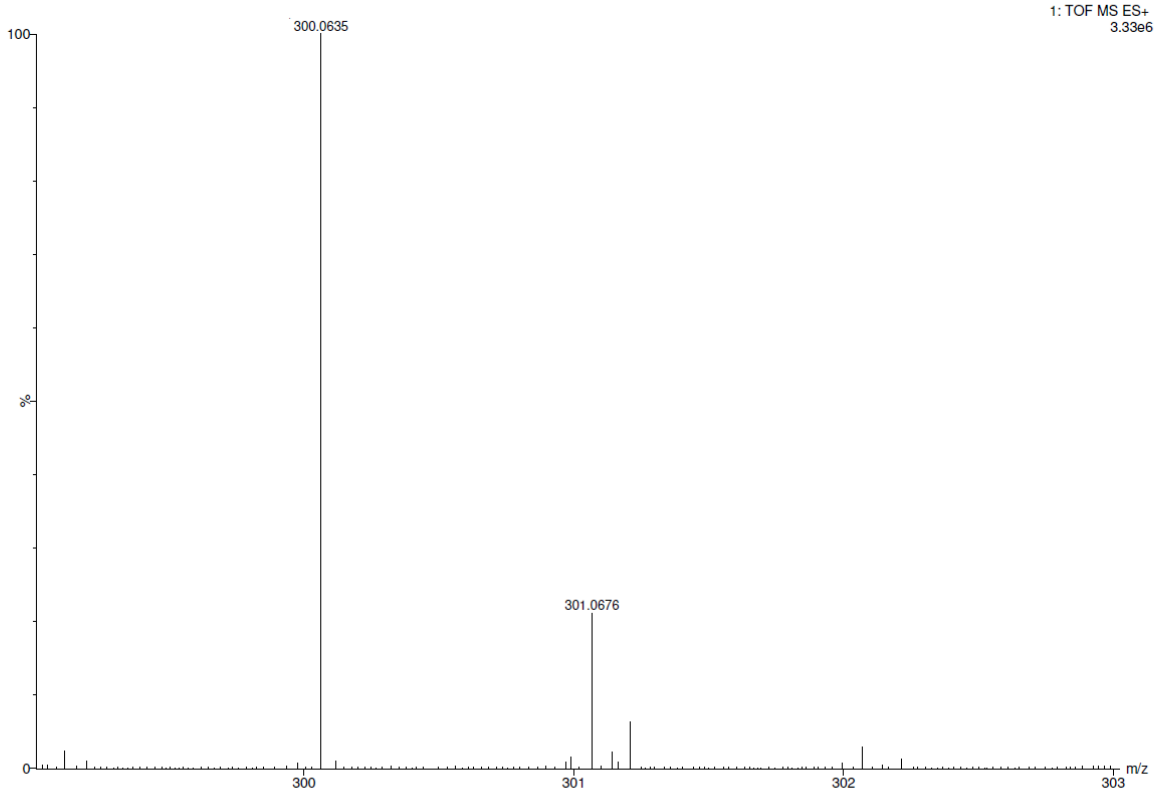
1: TOF MS ES+
3.33e6
300.0635
301.0676
100
%
0
300
301
302
303
m/z

3d. (*E*)-3-(4-(pyrrolidin-1-yl)phenyl)-1-(*p*-tolyl)prop-2-en-1-one.

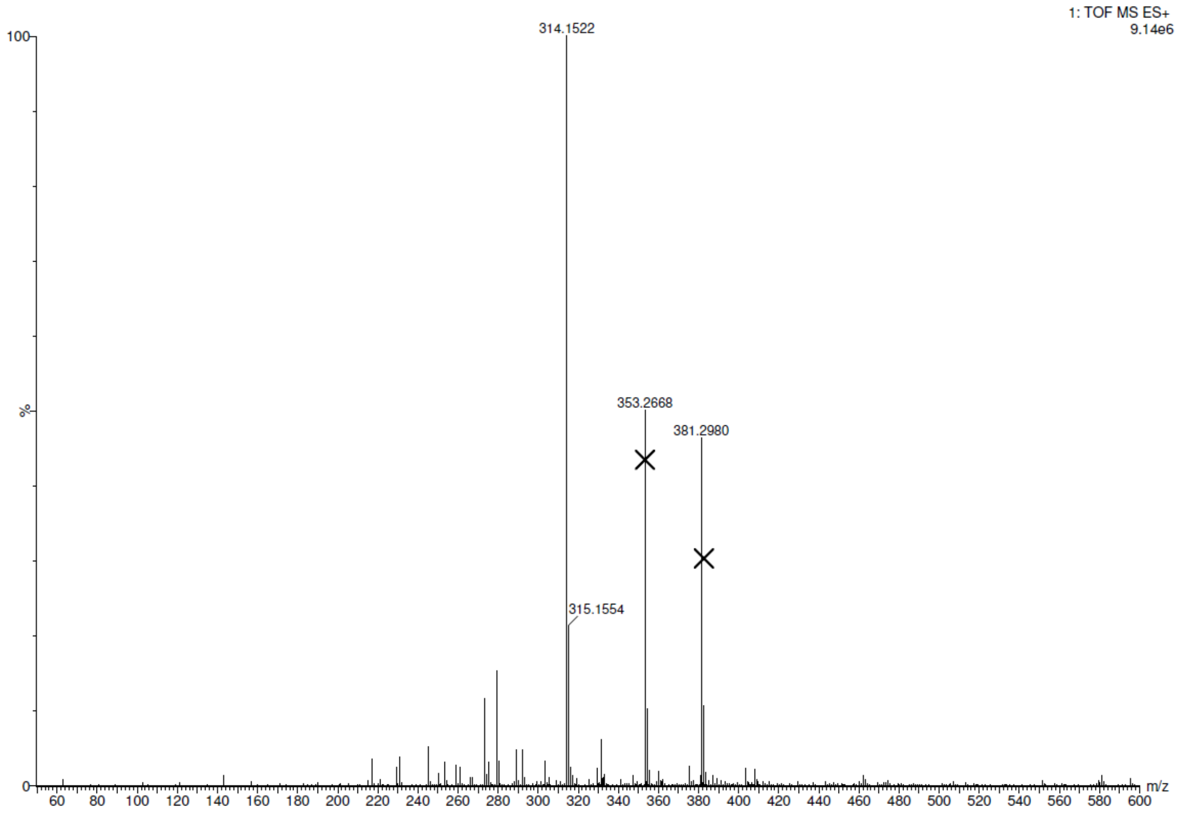

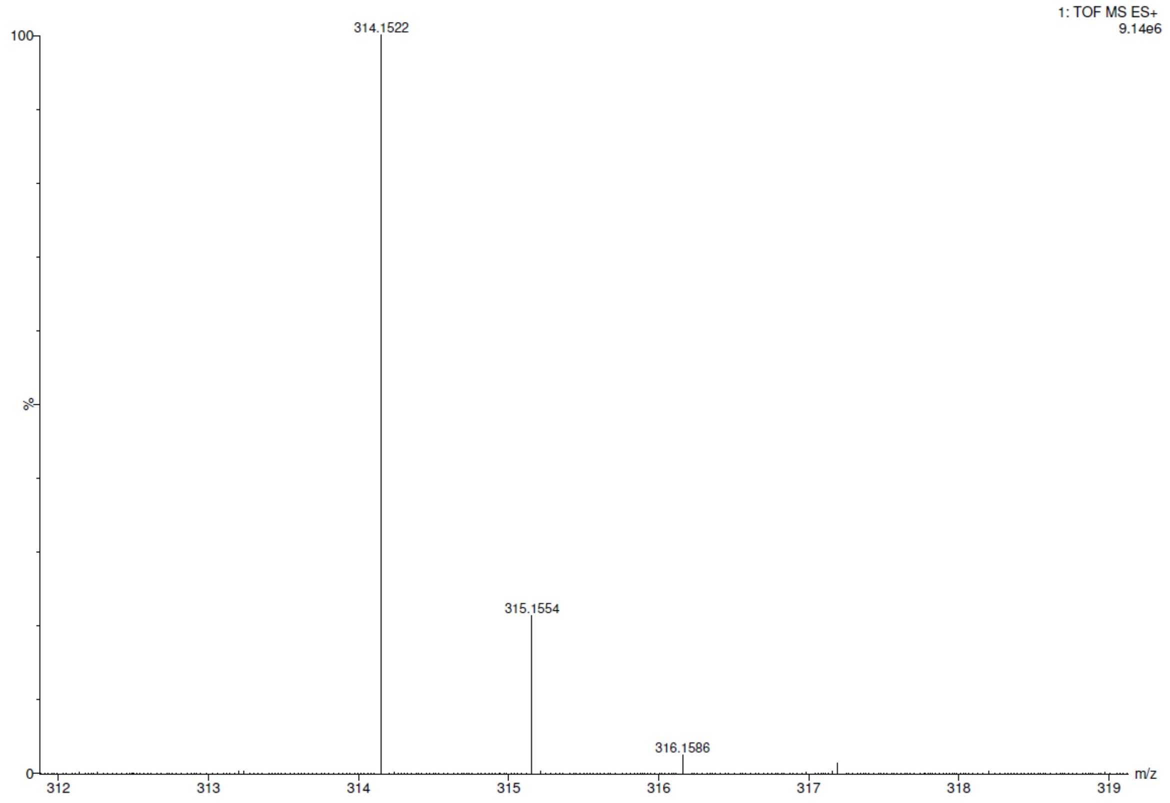

3e. (*E*)-3-(benzo[*d*][1,3]dioxol-5-yl)-1-(p-tolyl)prop-2-en-1-one.

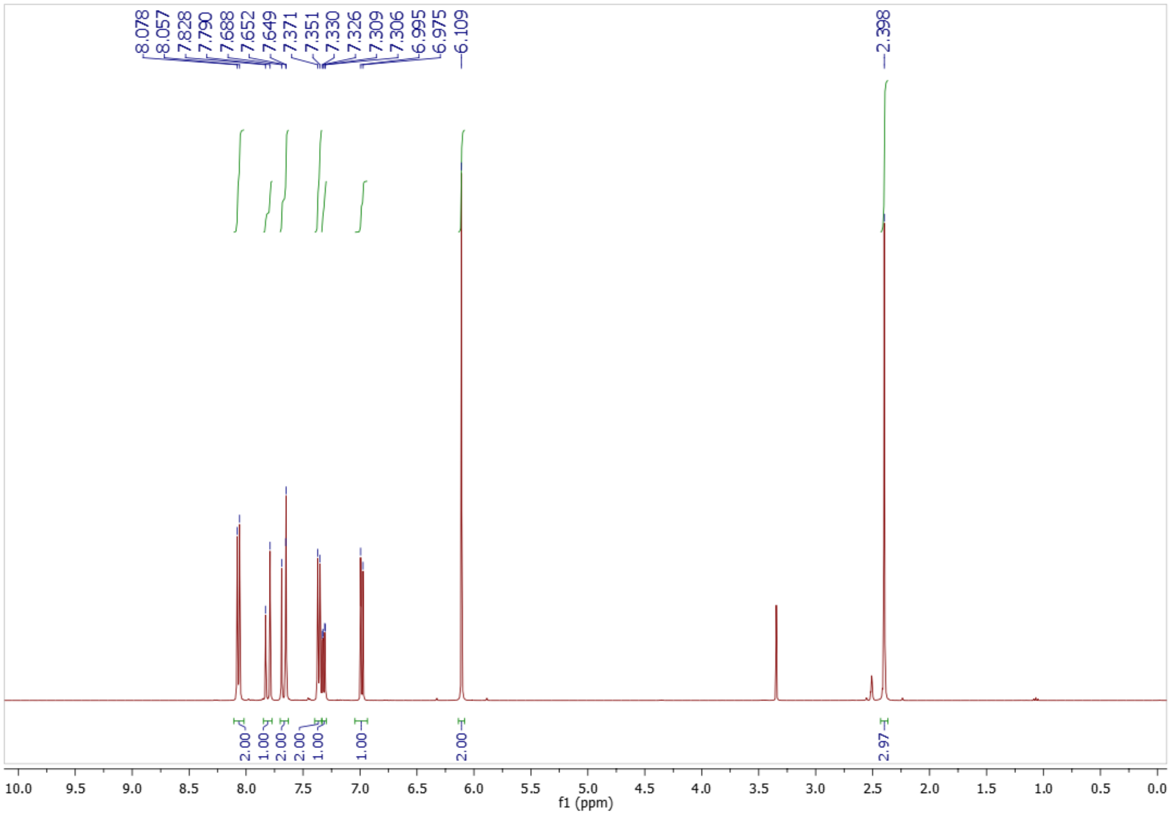

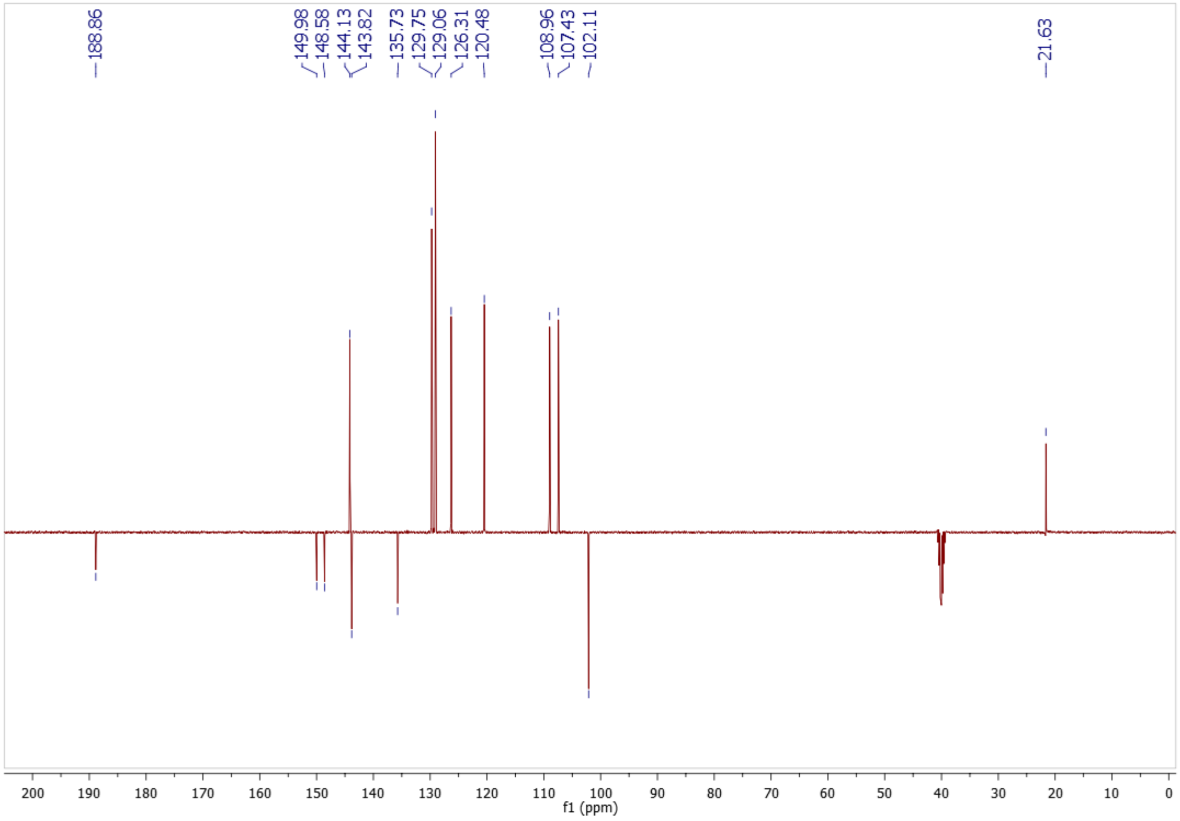

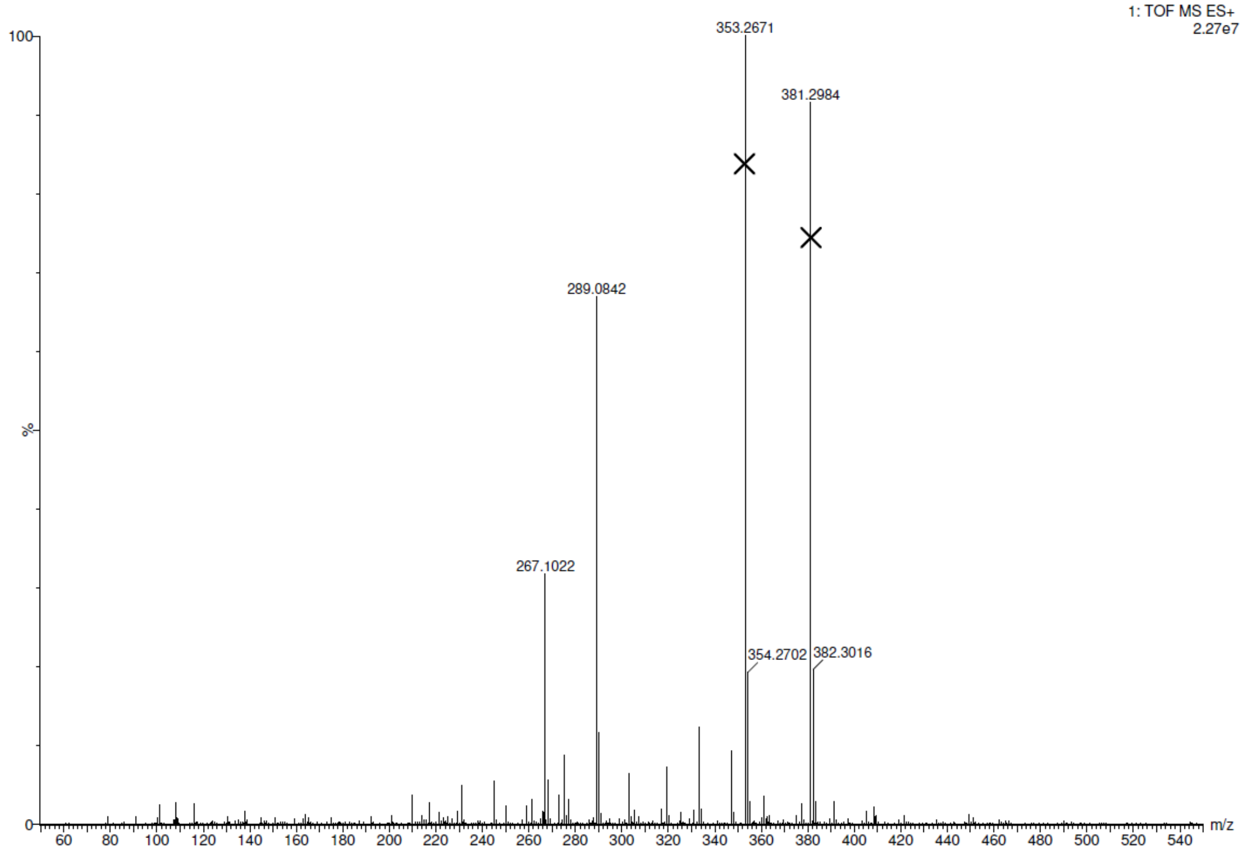
1: TOF MS ES+
2.27e7
353.2671
381.2984
289.0842
267.1022
354.2702
382.3016
%
m/z

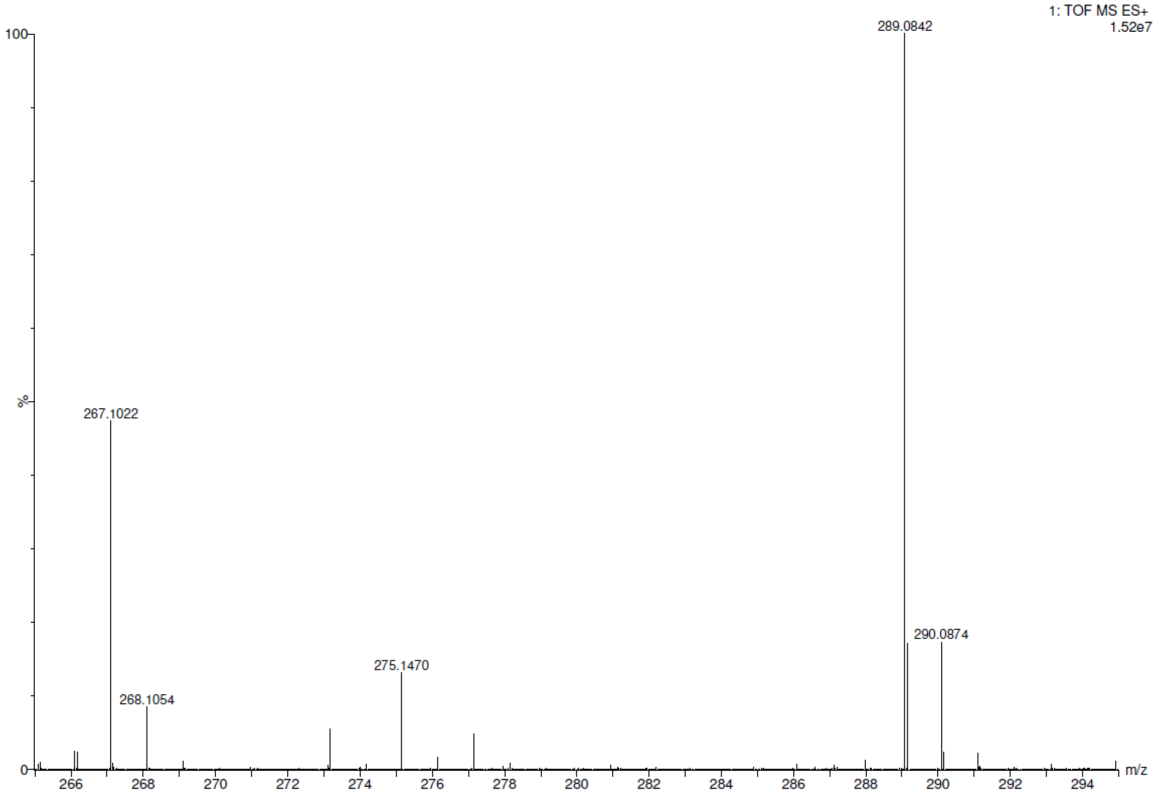
1: TOF MS ES+
1.52e7
289.0842
267.1022
290.0874
275.1470
268.1054
%
m/z

3f. (*E*)-4-(3-(4-(benzyloxy)phenyl)acryloyl)benzonitrile.

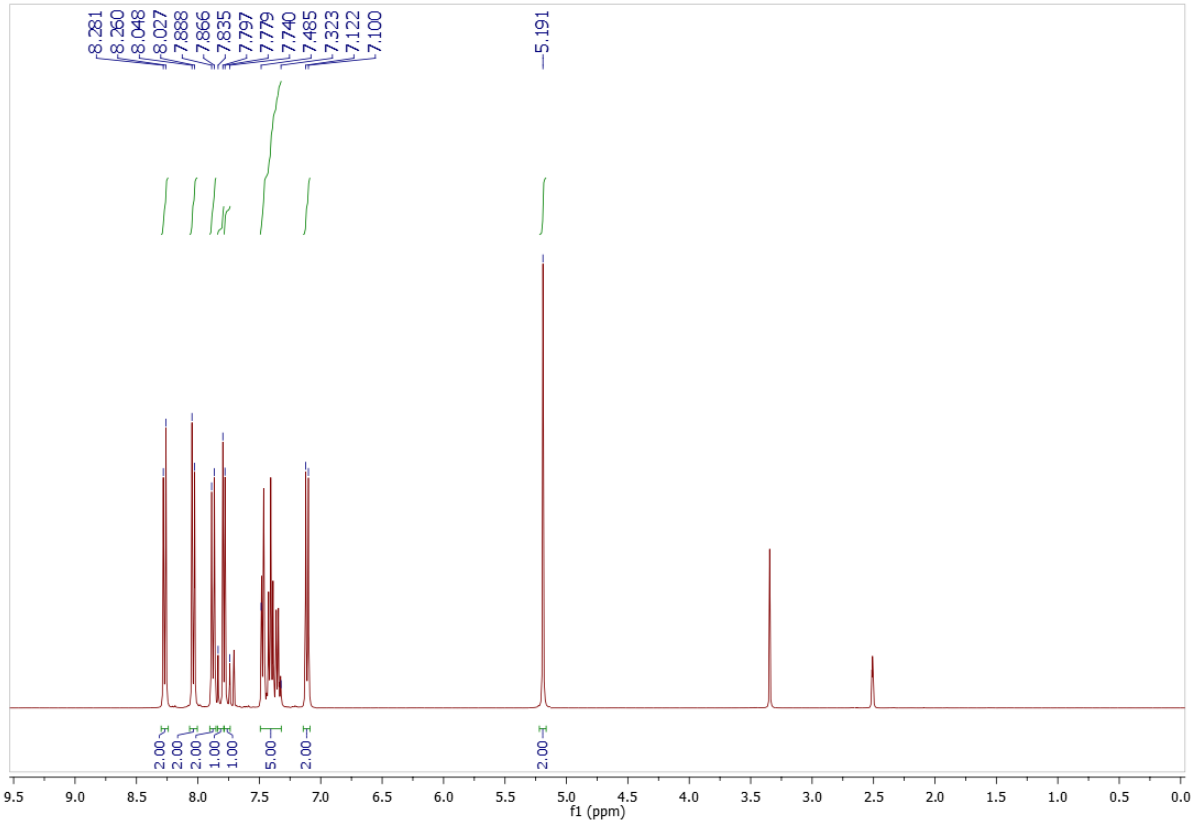

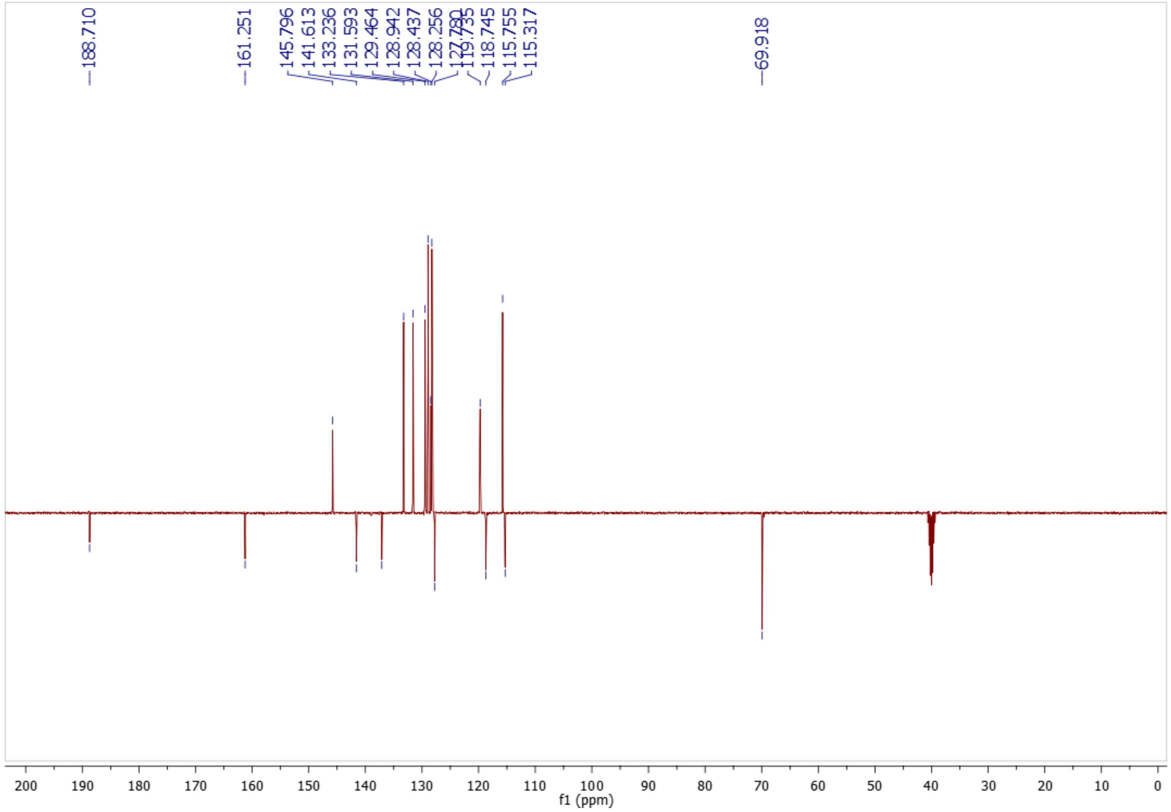

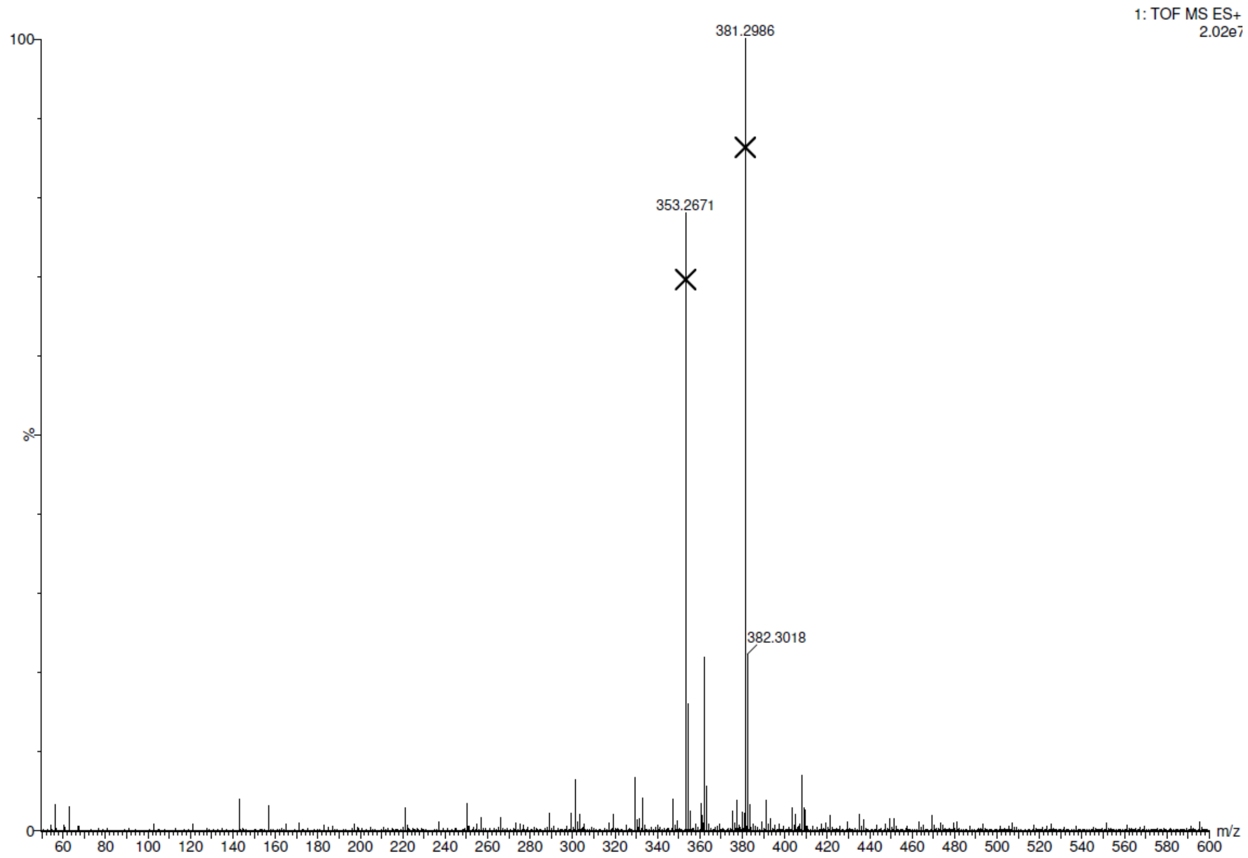
1: TOF MS ES+
2.02e7
381.2986
353.2671
382.3018
100
%
0
m/z
60
80
100
120
140
160
180
200
220
240
260
280
300
320
340
360
380
400
420
440
460
480
500
520
540
560
580
600

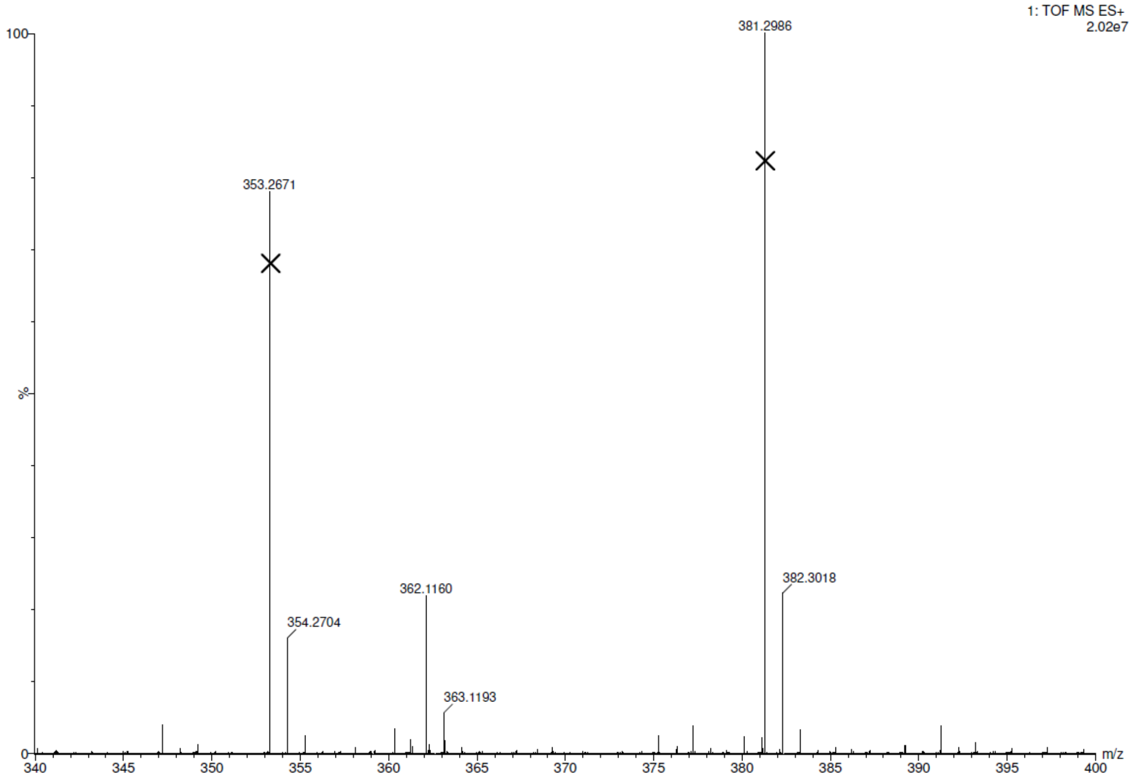
1: TOF MS ES+
2.02e7
381.2986
353.2671
362.1160
354.2704
363.1193
382.3018
100
%
0
m/z
340
345
350
355
360
365
370
375
380
385
390
395
400

3g. (*E*)-3-(4-(dimethylamino)phenyl)-1-phenylprop-2-en-1-one.

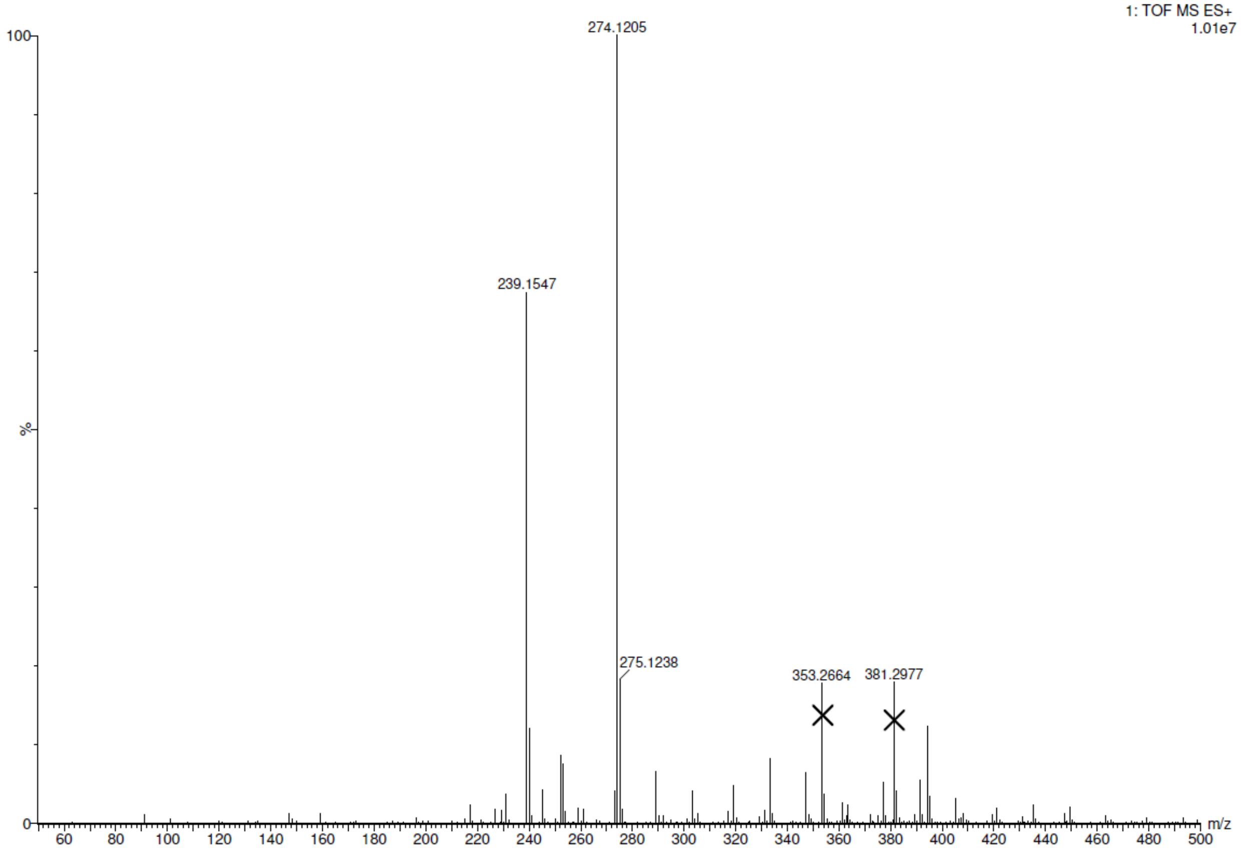

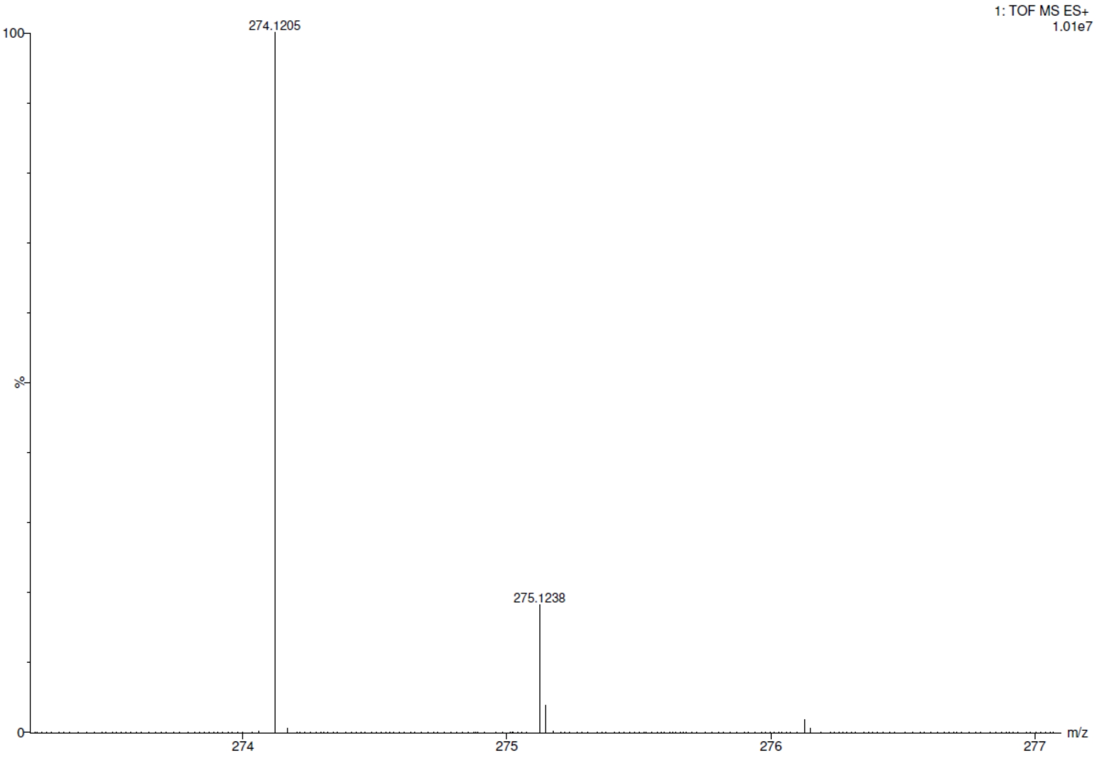

3h. (*E*)-3-(4-hydroxyphenyl)-1-(p-tolyl)prop-2-en-1-one.

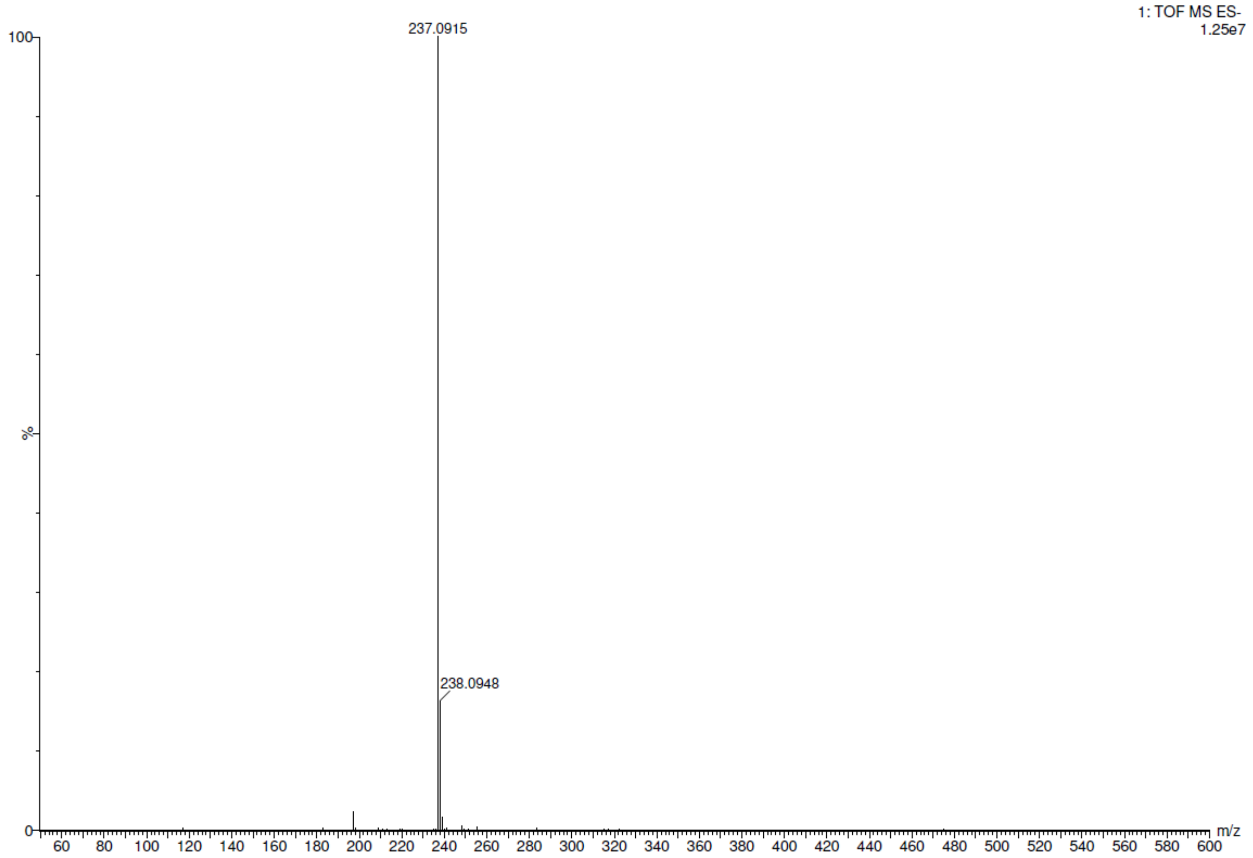

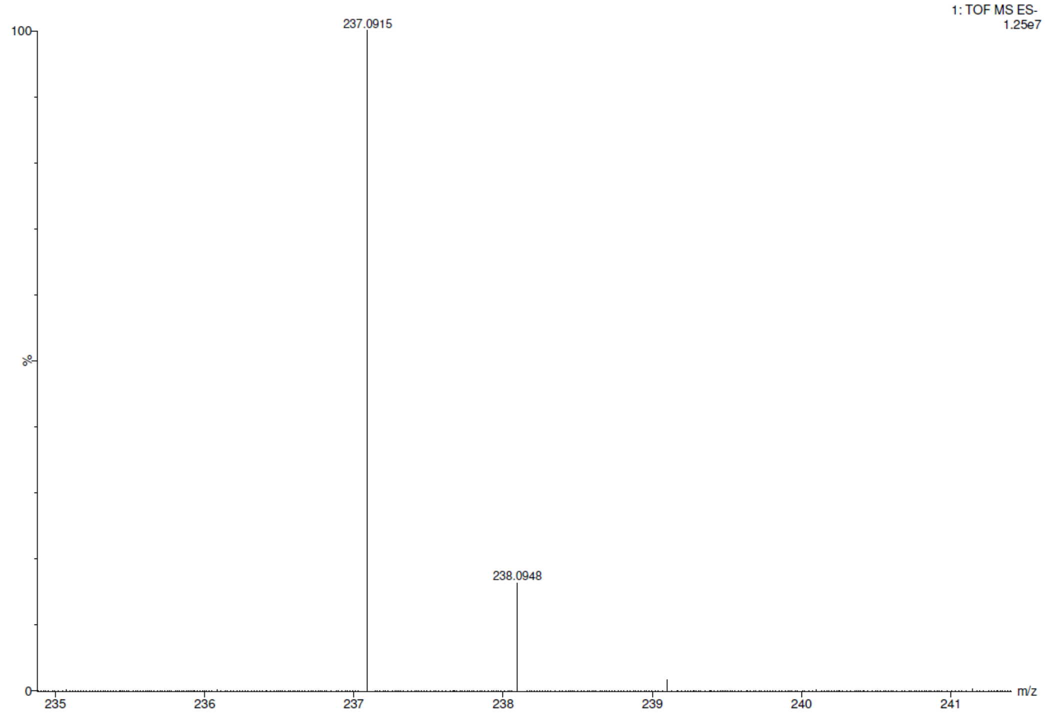

3i. (*E*)-3-(4-(1-phenylethoxy)phenyl)-1-(p-tolyl)prop-2-en-1-one.

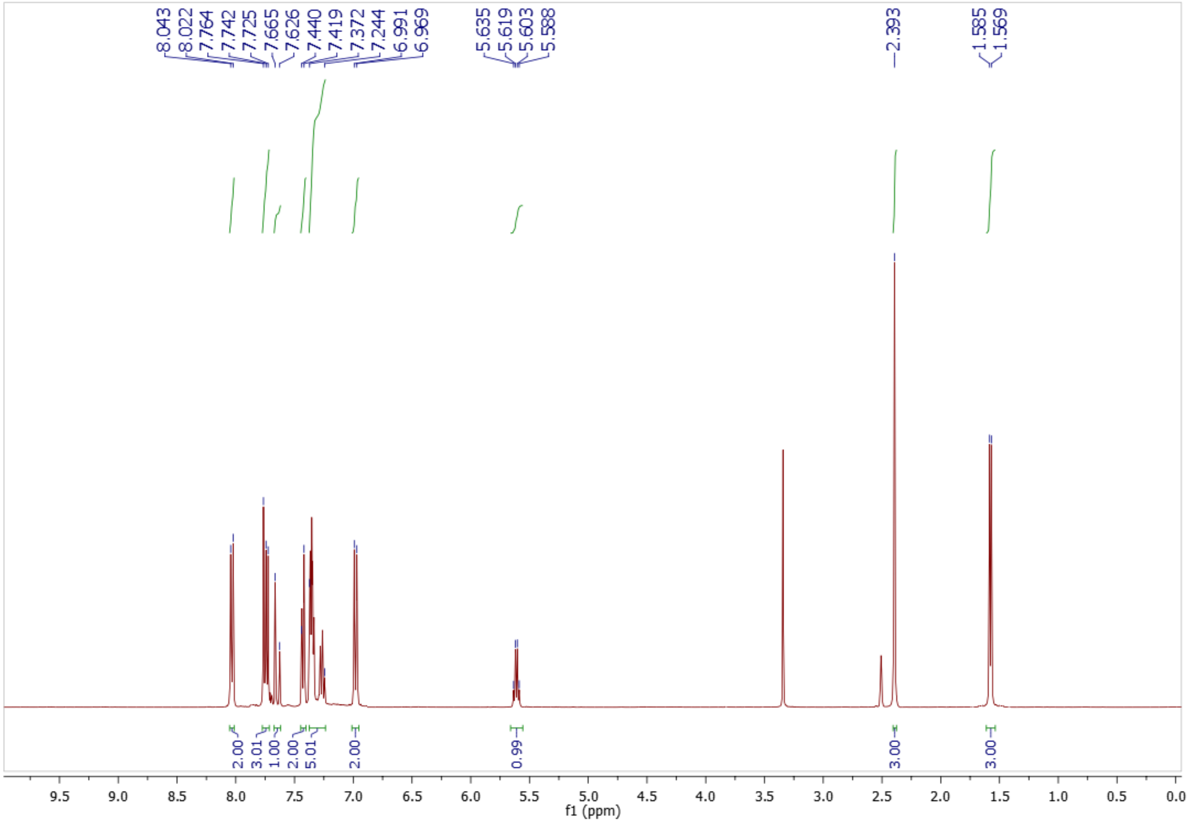

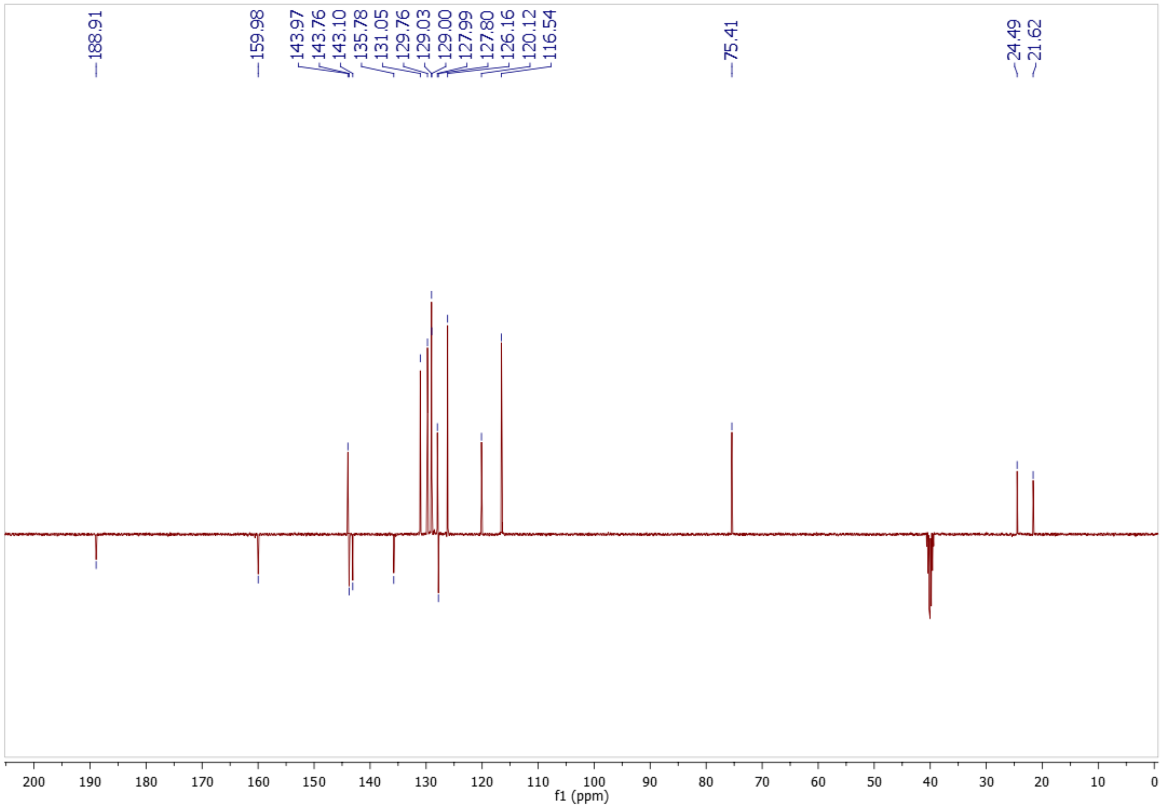

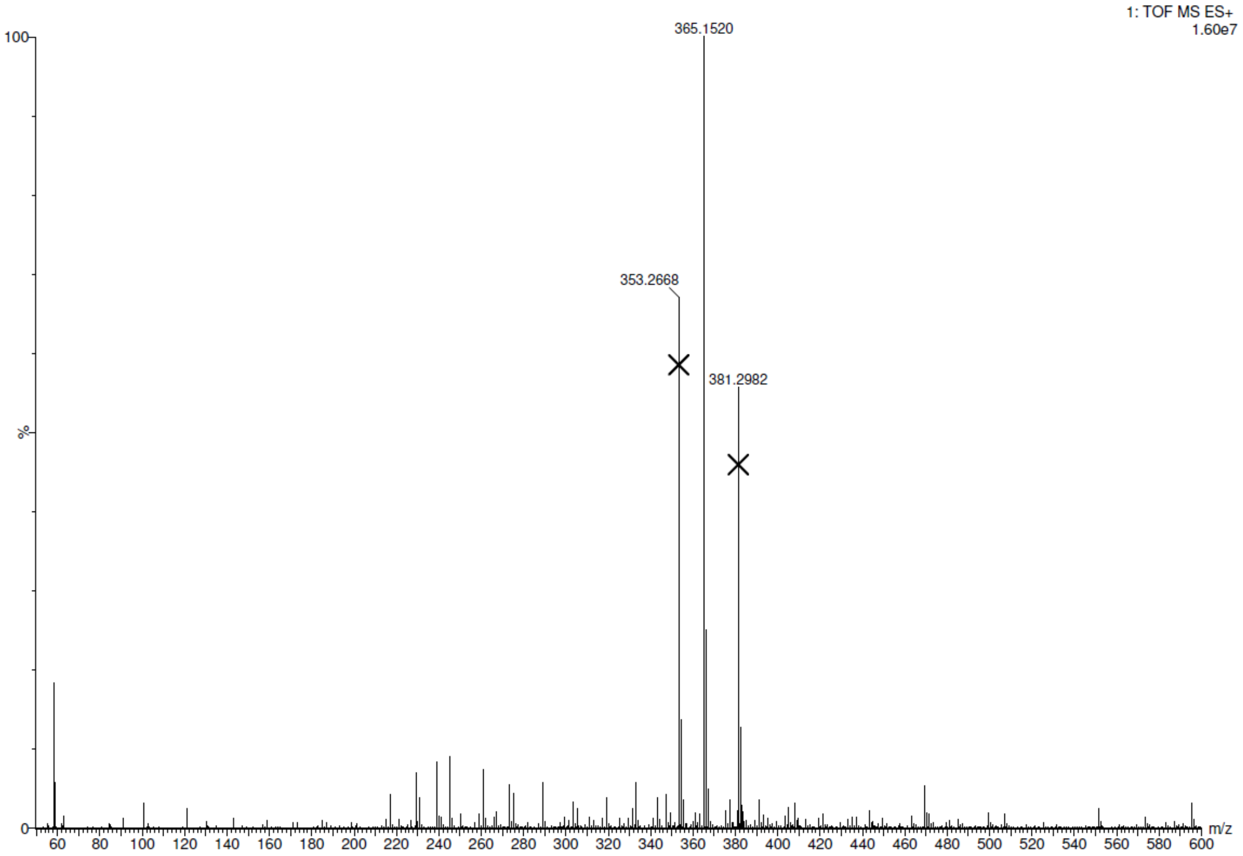
1: TOF MS ES+
1.60e7
365.1520
353.2668
381.2982
100
%
0
60
80
100
120
140
160
180
200
220
240
260
280
300
320
340
360
380
400
420
440
460
480
500
520
540
560
580
600
m/z

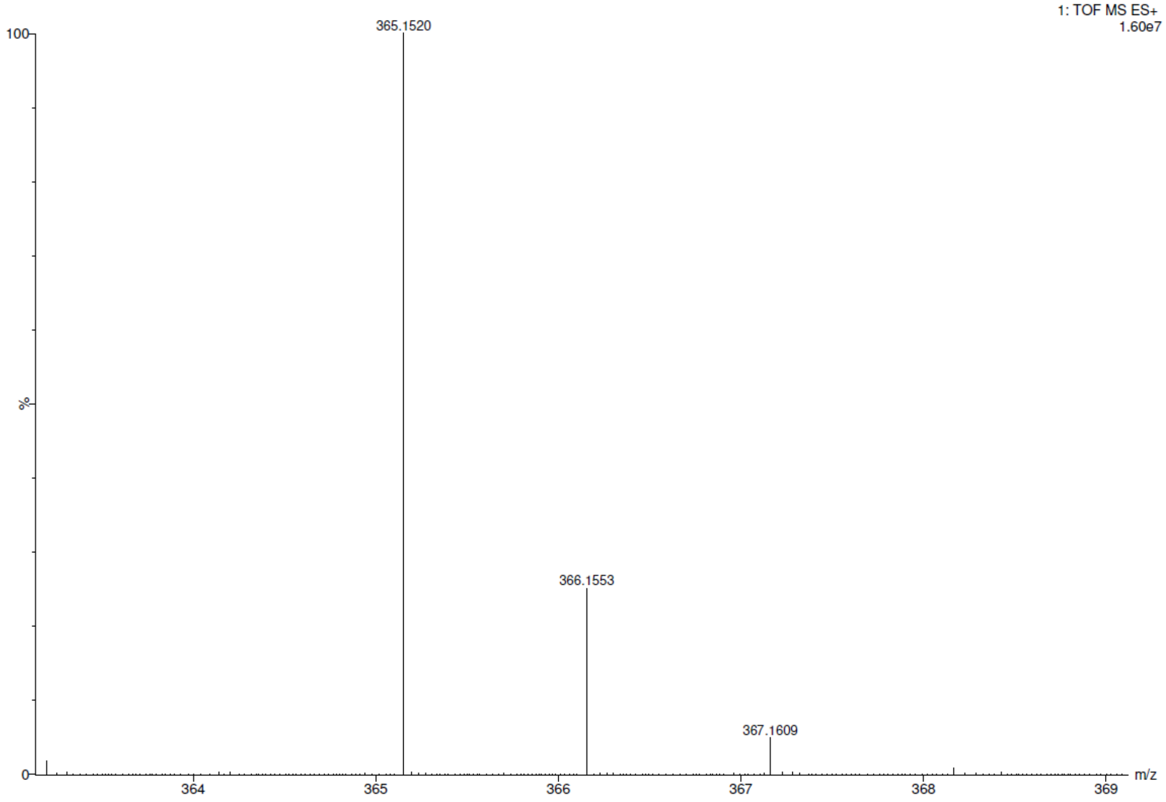
1: TOF MS ES+
1.60e7
365.1520
366.1553
367.1609
100
%
0
364
365
366
367
368
369
m/z

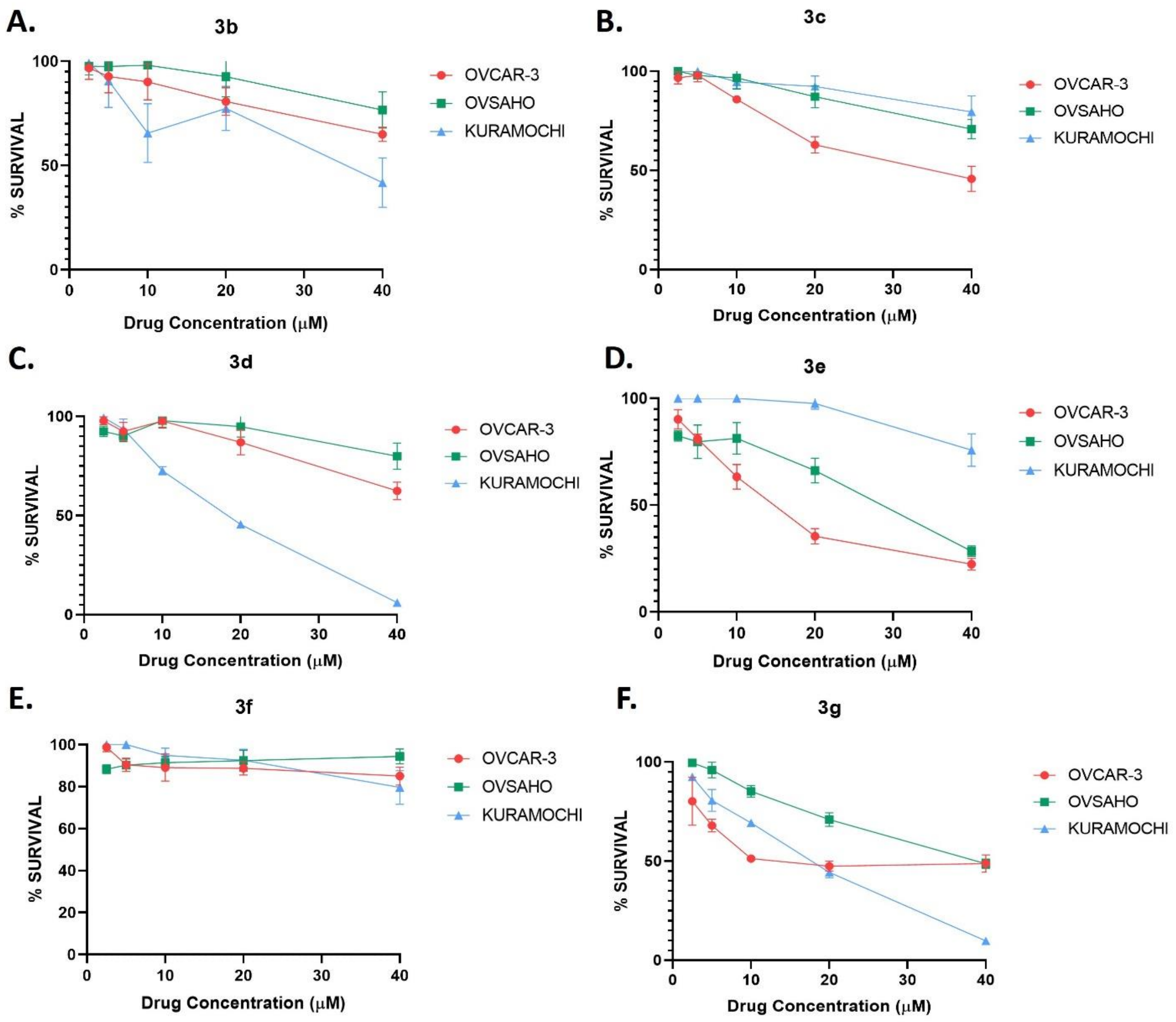

Figure S1. Cell viability analysis of compounds **3b(A), 3c(B), 3d(C), 3e(D), 3f(E) and 3g(F)** on OVCAR-3, OVSAHO, and KURAMOCHI cells. Cells were treated with increasing concentrations of the compounds (2.5 –40 μM) for 72 h. All results were normalized to data of negative control DMSO.
